# External immune sentinels in the seawater ecosystem: the specialization of bivalve hemocytes

**DOI:** 10.3389/fimmu.2026.1765948

**Published:** 2026-02-18

**Authors:** Magalí Rey-Campos, Amaro Saco, Antonella Panebianco, Judit Castro, Alejandro Romero, Beatriz Novoa, Antonio Figueras

**Affiliations:** Instituto de Investigaciones Marinas (IIM-CSIC), Vigo, Spain

**Keywords:** bivalves, evolutionary adaptation, external immunological system, immune sentinel cells (ISCs), neoplastic hemocytes

## Abstract

Immune sentinel cells in vertebrates play key roles in coordinating immune responses at the organism–environment interface. We describe a system in marine bivalves that could be analogous, with immune sentinel hemocytes (ISCs) that function on the surface of the organism but which could be specialized to act also outside the body. In bivalves, hemocytes migrate into the intervalvar cavity and surrounding seawater, where they remain viable, exhibit an immune-activated transcriptomic profile and tolerate acute infections. The key novel findings of mussel ISCs include: i) transfer between individuals, ii) apoptosis resistance, and iii) lack of allorecognition. Their transcriptomic profiles partially overlap with transmissible neoplastic hemocytes, but they retain a distinct functional immune identity. The functional characterization and visualization of ISCs in the marine ecosystem uncovers their potential for transfer between individuals. The description of this externalized immune defense system might constitute an evolutionary adaptation in marine invertebrates, advancing the understanding of population-level immunity.

## 1 Introduction

The interface between an organism and its environment is a critical zone for immune surveillance and defense, particularly in marine ecosystems characterized by high microbial diversity and the presence of potential pathogens. Marine invertebrates must continuously manage this microbial exposure while maintaining physiological homeostasis, a challenge that has driven the evolution of unique immunological adaptations.

In vertebrates, immune sentinel cells (ISCs) are well-characterized for their ability to detect pathogens and damage-associated molecular patterns (PAMPs and DAMPs). These cells coordinates early immune responses through the recruitment of effector cells and the production of pro-inflammatory mediators and antimicrobial peptides (1). The most studied vertebrate sentinel cells include macrophages and dendritic cells (2, 3), although fibroblasts (4, 5), neural stem cells (6), intestinal tuft cells (7), and skin-resident immune cells such as Langerhans cells and keratinocytes (8) also show sentinel functions. These cells are predominantly located in mucosal tissues, which constitute the interface with the environment (for instance, the mammalian intestine or skin). At this point, the ability to migrate and to enhance cell-to-cell communication is essential for early immune defense (9).

In contrast, the presence and function of analogous sentinel strategies in invertebrates, particularly at external surfaces, remain poorly defined. This gap is notable considering the role of many marine invertebrates as ecological keystone species. Their survival depends on a spectrum of environmental threats. The large sizes and wide distribution of certain invertebrates such as the sea star *Asterias amurensis*, the European green crab *Carcinus maenas* or mussels such as *Dreissena polymorpha* or *Mytilus* species (10), imply the existence of sophisticated and potentially unknown immune mechanisms.

Hemocytes are recognized as the principal immune cells in invertebrates (11–17), possessing migratory, phagocytic, and cytotoxic capacities (18–21), which resemble vertebrate macrophages. However, the key evolutionary question is whether invertebrate hemocytes fulfill specialized sentinel roles at the organism-environment interface.

Marine bivalves, particularly *Mytilus* species, provide a compelling model system for investigating the evolution of specialized immune cells in invertebrates. As sessile and filtering-feeding animals, they are continually exposed to high loads of environmental microbes and contaminants (22–24), displaying immunological robustness and ecological success. *Mytilus* species are known for their resistance (25) and their ability to adapt to highly diverse environments (26). These features are supported by unique genetic traits, such as the first open pan-genome described in a metazoan (*Mytilus galloprovincialis* (27)). Collectively, these characteristics suggest the evolution of unconventional immunological strategies.

While mucosal immunity is increasingly recognized in bivalves (28–30), their unique anatomy presents further opportunities for specialized immune interactions at the organism-environment boundary. Several fluid-filled compartments, including the paleal and intervalvar spaces (31, 32), have been identified as sites where immune cells migrate, though their implications in immune surveillance remain poorly understood (29, 33, 34). Bivalves often have their shells open for feeding and respiration. Therefore, direct interaction between the hemocytes and the external marine environment is probable. These interfaces represent the frontline where interactions with environmental stressors, microorganisms and contaminants occur (22–24). Some evolutionary questions arise on whether marine invertebrates have developed surveillance systems that operate even between individuals.

Several lines of evidence suggest functional specialization of marine invertebrate hemocytes, including their active migration dynamics (19, 33–35), their activation after physical alerts such as changes in temperature (33), tissue injuries and shell repair (18, 34), and also after bacterial infections (12, 36). While the migration of hemocytes could favor the spread of pathogens (33, 35), an alternative host-adaptive evolutionary perspective has not been fully explored. The ability to move into the external environment and survive in seawater, even entering in other individuals, also parallels, at a mechanistic level, the transmissible neoplasia cancer propagation in bivalves (37, 38), suggesting mechanisms for extracorporeal cell survival and transfer.

Our previous work demonstrated that intervalvar mussel seawater contains functional and active hemocytes that exhibit distinct characteristics compared to internal hemocytes, including smaller size, enhanced migration, and an activated immune gene expression profile (e.g., IL17 overexpression) (34). This suggested they could be the first immune barrier in mussels (*M. galloprovincialis*). These cells, which we refer to interchangeably as “intervalvar cells” (to highlight that they are specifically found in the intervalvar cavity) or “external hemocytes” (to expand the concept towards the movement to the medium and reaching other individuals) exhibit specialized immune functions, leading us to designate them as “immune sentinel cells (ISCs)”. Therefore, the presence of these viable, transcriptionally active cells in the intervalvar seawater suggests that bivalves could possess external immune sentinels to detect environmental threats. However, a fundamental question remained to be solved: do these cells constitute an evolutionarily specialized lineage of immune sentinels? In this work, we characterized bivalve ISCs, suggesting that they could represent an extracorporeal immune sentinel system. Although further work is needed to complete the characterization, this may serve as a baseline for investigating functional insights of externalized immunity.

## 2 Materials and methods

### 2.1 Experimental design

Adult mussels (*Mytilus galloprovincialis*), clams (*Ruditapes philippinarum*) and cockles (*Cerastoderma edule*) were obtained from a commercial shellfish farm (Vigo, Galicia, Spain). Shells were cleaned of environmental residues, and they were acclimatized for one week in open-circuit seawater tanks (two animals/L) at 15 °C with aeration. The animals were fed daily with the algae *Phaeodactylum tricornutum* and *Isochrysis galbana* (20,000 cells/mL). Bivalves were kept without food for 24 h prior to the experiments to avoid interference from algae.

The RNA-seq experiment consisted in expose mussels to a *Vibrio* sp*lendidus* waterborne infection. We used this bacterium because it causes high mortalities in farmed molluscs (39) and we previously demonstrated that a potent immune stimulation occurs in mussels (13, 30). The bacterial load in the tank was 10^5^ CFU/mL. Twenty-four hours after the exposure, samples of intervalvar hemocytes, hemolymph hemocytes, and gills were collected.

Samples were pooled from six individuals, to ensure sufficient RNA in the intervalvar seawater samples. Subsequently, the cellular samples were centrifuged at 4 °C for 10 min at 2,500 g, and the pellet was resuspended in 200 μL of homogenization buffer for RNA extraction. Gills were physically homogenized using the same buffer. All the samples were processed under identical experimental conditions to minimize batch effects, and were stored at -80 °C until RNA isolation.

### 2.2 IL17 antibody synthesis

Interleukin-17 is a proinflammatory cytokine that is highly expanded in marine molluscs and echinoderm species. Mussels have 23 subfamilies of IL17, which are conserved among individuals and shared between closely related *Mytilidae* species. Certain isoforms, such as IL17-3, are explicitly implicated in inflammatory and mucosal responses (40). Based on our evolutionary study of IL17 (40), we were able to synthesize a rabbit polyclonal antibody specific for mussels. The synthesis was performed by GenScript Biotech (Netherlands). The IL17 peptide sequence used for this purpose was PIQQKIQVLKRAGC. Two rabbits were immunized with the peptide to produce antibody-enriched serum.

### 2.3 Immunofluorescence of ISCs and cell transfer experiments

Intervalvar seawater and hemolymph were collected from mussels, clams and cockles. Intervalvar seawater was sampled by draining it into a Petri dish, slightly separating the valves with a disinfected spatula, and then filtered with a 40 µm filter. A notch was made in the shell to extract the hemolymph from the adductor muscle with a syringe equipped with a 25-gauge needle.

Once the mussel samples were obtained, the hemocytes were placed in a 24-well plate and kept at 15 °C for 30 min until they settled onto the glass covers at the bottom. After this, the hemocytes were fixed in 4% paraformaldehyde (PFA) for 10 min at 4 °C, and nonspecific sites were blocked with PBS + 0.1% saponin + 2% BSA for 60 min at room temperature. Next, mussel hemocytes were incubated overnight (4 °C) with a rabbit polyclonal anti-myticin C antibody (1:50) (41) or with a rabbit polyclonal anti-interleukin-17 antibody (1:500). Alexa Fluor 568 goat anti-rabbit (1:500; Life Technologies, Carlsbad, CA, USA) was used as secondary antibody. Hemocytes were also stained with Alexa Fluor 488 phalloidin (Invitrogen, Carlsbad, CA, USA) and 1 µg/mL 4′,6-diamidino-2-phenylindole (DAPI; Invitrogen, Carlsbad, CA, USA). Samples were mounted on slides using ProLong Gold reagent (Invitrogen, Carlsbad, CA, USA) and visualized on an SPE confocal microscope (Leica, Wetzlar, Germany).

Staining was also done in clam and cockle hemocytes to compare the morphology and structure of intervalvar hemocytes from mussels to other bivalves.

Because hemocytes moves to the intervalvar space in bivalves, we wanted to determine if they spread to the external environment, seawater, and if the hemocytes manage to enter other animals. For this purpose, mussel cohabitation experiments were done. To set up the experimental conditions, several tests were conducted by labeling the mussel hemocytes with Vybrant™ CFDA solution (Life Technologies, Carlsbad, CA, USA). This cell labeling kit is used as a long-term cell marker. We tested two concentrations *in vitro* (10 µM and 20 µM). After confirming that the cell labeling was nearly 100%, we tested the staining efficiency *in vivo*, by injecting several volumes of the reagent (100 µL, 500 µL and 1 mL) and sampling the hemocytes 1 h, 5 h and 24 h after the staining. Finally, after testing the hemocyte dynamics at 1 h, 3 h, 5 h and 24 h, the higher transfer rates were detected at 5 h. Therefore, two animals were placed in the same tank for 5 h to follow the transfer of hemocytes. One of the animals was injected in the adductor muscle with 1mL of Vybrant™ CFDA solution (Life Technologies, Carlsbad, CA, USA, 20 µM). After 30 min out of the seawater, the mussel with labeled cells (source mussel) and a naive mussel (receptor mussel) were placed in the same tank for 5 h. After that, the intervalvar cells and hemolymph cells of both animals were collected for fixation, and myticin C was immunostained and visualized using a confocal microscope (the fixation, antibody immunolabeling and visualization procedures were the same as described above). The fluorescent visualization of hemocytes from the intervalvar space and hemolymph of the two animals (donor and recipient) made it possible to estimate the percentage of cells transfer between individuals. The total number of cells assessed was 160,000 in each case. This experiment was repeated three times, and three cohabitation pairs were used in each attempt. Green labeled hemocytes (i.e., prior stained with the Vybrant™ CFDA SE cell kit) were found in both the source and the receptor mussels.

### 2.4 Determination of hemocytes allorecognition

The degree of allorecognition of mussel hemocytes has been assessed by myticin C gene expression, myticin C immunofluorescence, and reactive oxygen species production (ROS). Hemocytes from mussels were mixed in pairs for 1 h to be further run through the four immunological assays. The first one was a qPCR of the immunological marker myticin C. Hemocytes from 10 individual mussels were mixed in pairs for 1 h. Then, the samples were centrifuged at 4 °C for 10 min at 2,500 g, and the pellet was resuspended in 200 μL of homogenization buffer for RNA extraction. After obtaining the RNA (the procedure is the same as described in the RNA isolation section), cDNA was produced using the NZY First-Strand cDNA Synthesis Kit (NZYtech). Specific primers for the detection of myticin C were used (F: ATTTGCTACTGCCTTCATTG and R: TCCATCTCGTTGTTCTTGTC). Gene expression was analyzed by quantitative PCR (qPCR) using the StepOnePlus real-time PCR system (Applied Biosystems). Standard cycling conditions were 95°C for 10 min, 40 cycles of 95°C for 15 s and 60°C for 30 s. All reactions were performed with technical duplicates. Relative gene expression levels were calculated using the 18S ribosomal RNA as gene reference (F: GTACAAAGGGCAGGGACGTA; and R: CTCCTTCGTGCTAGGGATTG), following the method described by Pfaffl (42).

Furthermore, following the same experimental procedure of comparing hemocytes from only one individual with hemocytes after being in contact with those hemocytes from another animal, we performed an immunofluorescence using the myticin C antibody (the same procedure described in previous immunofluorescence section). After that, we measured the quantity of fluorescence by flow cytometry with the FACSCalibur cytometer (BD Biosciences). Data analyses were performed using CellQuest software (BD Biosciences). The same was done using zymosan A (Sigma Aldrich) as a phagocytosis inducer before the immunostaining to determine if, in an immunologically activated state, hemocytes could be more responsive to the interaction with hemocytes from other individuals.

Finally, ROS production was tested in the hemocytes of individual mussels and hemocytes from pairs of individuals. After 1 h of interaction, the production of reactive oxygen species was analyzed using the luminol-enhanced chemiluminescence (LC) method. One hundred microliters of cell suspension were plated (four technical replicates) in a white opaque 96-well flat microplate (Thermo Fisher) and incubated for 1 h at 15 °C. Zymosan A (Sigma Aldrich) was used to trigger ROS production, and luminol (5-amino-2,3-dihydro-1,4-phthalazindione) (Sigma Aldrich) was used as a light emitter. Cells from 8 animals were tested separately and then mixed in pairs. A 0.1 M luminol solution in dimethyl sulfoxide (DMSO, Sigma Aldrich) was diluted in FSW to obtain a final concentration of 10 mM. Zymosan A was diluted in the luminol solution to a final concentration of 1 mg/mL. One hundred microliters of zymosan A were added to each well. The generation of relative luminescence units (RLU) was measured using a Glomax Discover Microplate device (Promega) eight times at 5-minute intervals.

### 2.5 RNA isolation and Illumina sequencing

RNA isolation was performed with the Maxwell RSC simplyRNA Kit (Promega, Madison, WI, USA), according to the manufacturer’s instructions. The RNA concentration was measured using the NanoDrop spectrophotometer (NanoDrop Technologies, Inc., Wilmington, DE, USA). According to the RNA quantity, 3 control and 3 infected samples were selected for each of the 3 sample types. The selected samples were sequenced using NGS Illumina NovaSeq 6000 at Macrogen Inc. Korea (Seoul, Republic of Korea).

### 2.6 RNA-seq, differential expression and enrichment analysis

Raw sequencing reads were trimmed to remove adapter sequences and low-quality reads (with a quality score limit of 0.05). RNA-seq analysis was performed by mapping the paired and trimmed reads from each sample to the mussel genome (27), considering a length fraction of 0.8 and a similarity fraction of 0.8. A differential expression analysis was performed to compare gene expression levels and identify differentially expressed genes (DEGs). Genes with an absolute log2 fold change ≥ 1 and an adjusted p-value < 0.05 (Benjamini–Hochberg test) were considered differentially expressed. RNA-seq analyses were performed using the QIAGEN CLC Genomics Workbench, version 23 (https://digitalinsights.qiagen.com) and differential expression analyses were performed using DESeq2 (43).

Several enrichment analyses were performed to determine which biological processes were overrepresented in differentially expressed genes. The annotated mussel genome was used as a reference (27). Fisher’s exact tests were performed using a p-value of 0.05. These analyses were run using the OmicsBox Software (https://www.biobam.com/omicsbox).

In addition, a meta-analysis comparing the expression of intervalvar hemocytes, hemolymph hemocytes and MtrBTN hemocytes (transmissible cancer cells) was also done using data available in public databases. Control hemolymph hemocytes and intervalvar hemocytes from the current work were used, in addition to *M. edulis* hemolymph hemocytes and MtrBTN cells from previous work (PRJNA749800) (44). All samples were mapped to the same *M. galloprovincialis* reference (27) with equivalent mapping rates between the two *Mytilus* species. Several analyses were performed to get similar and distinctive expressed genes and pathways between the hemocytes found in intervalvar cavity and mussel neoplastic hemocytes (**Supplementary Figure 1**). The common DEGs shared by intervalvar hemocytes and mussel neoplastic hemocytes were detected by performing a differential expression analysis (DESeq2 (43)) of these grouped samples against the group of hemolymph hemocytes samples. DEGs modulated between neoplastic hemocytes and intervalvar hemocytes were discarded (Method 1). The alternative method (Method 2) consisted in analyzing separately the modulation of neoplastic hemocytes and intervalvar hemocytes against hemolymph hemocytes and retrieving the genes modulated in both cases. After that, DEGs also modulated between intervalvar hemocytes and neoplastic hemocytes were removed. The set of genes found with method 2 was smaller and almost entirely contained within the set from method 1. Furthermore, the differences between neoplastic hemocytes and intervalvar hemocytes were also analyzed by retrieving the DEGs that were modulated between those two samples and also with respect to internal hemocytes (**Supplementary Figure 1**).

### 2.7 HSP70 analysis

The proteomes encoded in several metazoan genomes were downloaded from NCBI and a species tree was made using the Python BUSCO_phylogenomics script (45) and ASTRAL (46). These proteomes were subjected to an orthology analysis using Orthofinder (47) and HSP70 proteins were identified based on the presence of the Pfam domain. A phylogenetic analysis of HSP70 genes in mussels was performed using PHYML (48) with automatic evolutionary model detection. The resulting tree was annotated with iTOL (49) using the orthology information from the metazoan level analysis and the gene expression data comparing intervalvar and hemolymph hemocytes.

### 2.8 Microbiome characterization

We analyzed the presence of eukaryote and prokaryote sequences in intervalvar cells, internal hemocytes and gills. We mapped all the reads to reference databases, including eukaryotic and prokaryotic genome assemblies, using the CLC Microbial Genomics Module software (QIAGEN, Aarhus, Denmark). Before the taxonomic classification, reads were subsampled to 20 million for comparative purposes. Host-specific reads were filtered by mapping to the mussel genome (27). Information about reference databases used in this work is available in **Supplementary Table 1**. The mapping parameters used were: length fraction = 0.5, similarity fraction = 0.8 and a minimum seed length of 30.

To characterize the intervalvar virome, as well as that of the hemolymph and gills, reads that did not map to the mussel genome were assembled using the QIAGEN CLC Genomics Workbench, version 23 (https://digitalinsights.qiagen.com). After that, the contig list was filtered to discard sequences shorter than 1,000 bp. Viral identification-specific software was used to find viral contigs. The software used were Vibrant (50), VirSorter2 (51) and DeepVirFinder (52). After identifying viral contigs, a binning procedure was performed using the vRhyme software (53), with the composite dereplication method that generates new contigs from overlapping contigs. Finally, taxonomic classification was done with geNomad (54) when possible. A schematic of the virome analysis pipeline is provided in **Supplementary Figure 2**.

### 2.9 Bacterial load validation

Samples of mussel intervalvar seawater and hemolymph were seeded on Tryptic Soy Agar (TSA) plates (1.5% NaCl at 22 °C for 24 h) to count the number of colony-forming units per mL (CFU/mL). Five mussels were tested, and serial dilutions of all samples were performed and seeded in triplicate.

### 2.10 Apoptosis assay

An apoptosis assay was done using flow cytometry. The Invitrogen™ Annexin V Ready Flow Conjugates for Apoptosis Detection kit was used following the manufacturer’s instructions. Hemolymph and intervalvar seawater samples from 12 mussels were taken and diluted 1:1 in filtered seawater to avoid hemocyte aggregation. Hemocytes from each sample were then plated in 24-well plates and treated with UV (30 min) to induce apoptosis. Hemocytes of the same individuals were used as controls. Hemocytes were incubated for 1 h at 15 °C. After incubation, hemocytes were harvested and centrifuged at 4 °C for 10 min and at 300 g. The supernatant was removed, and 100 µL of annexin-binding buffer was added to prepare hemocyte samples for flow cytometry. Then, hemocytes were double stained with Annexin V-FITC and eBioscience™ 7-AAD Viability Staining Solution (Invitrogen™) to detect apoptotic and non-viable hemocytes, respectively, for 15 min at room temperature. Stained hemocytes were analyzed by flow cytometry with the FACSCalibur cytometer (BD Biosciences). Data analyses were performed using CellQuest software (BD Biosciences). The experiment was performed twice. An average of 5,300 events per sample were analyzed and separated according to forward scatter (FSC) and side scatter (SSC) parameters. A threshold FSC value of 20 was set to exclude sample debris, and a region of interest was delimited. The FL1 detector (λem = 450 nm) was used for apoptotic hemocytes and the FL3 detector (λem = 575 nm) was used for necrotic hemocytes.

The percentages of apoptotic and non-apoptotic hemocytes, as well as the morphology of control and UV-treated hemocytes, were also analyzed by microscopy. In this case, hemolymph and intervalvar seawater were extracted from 3 individuals. Hemocytes were treated with ultraviolet light for 30 min and were distributed on 50 mm FluoroDish Cell Culture Dish plates (World Precision Instrument). After treatment, the hemocytes were incubated for 1 h and then fixed using 4% paraformaldehyde (PFA) for 10 min. Hemocytes were also stained with 1 μg/mL of 4′,6-diamidino-2-phenylindole (DAPI) and visualized on a Leica DMi8 fluorescence microscope (Leica). Images were taken at 40X magnification, and the percentage of apoptotic and non-apoptotic hemocytes was counted in 3 images (332 µm x 332 µm) per individual.

### 2.11 qPCR for the assessment of apoptosis in ISCs

Differences in gene expression of apoptotic genes between hemocytes collected from the intervalvar cavity and hemolymph hemocytes of 5 mussels were confirmed by qPCR. The sampling procedure was the same as described in the previous section on apoptosis. RNA isolation was performed with the Maxwell RSC simplyRNA Kit (Promega, Madison, WI, USA) according to the manufacturer’s instructions. RNA concentration and purity were measured using a NanoDrop ND1000 spectrophotometer (NanoDrop Technologies, Inc., Wilmington, DE, USA). The RNA concentration was adjusted to 50 ng/mL, and cDNA was produced using the NZY first-strand cDNA synthesis kit (NZYtech). Specific primers were used to analyze caspase 1, caspase 2, caspase 3/7, caspase 8, Bcl2, Bax2 and 18S rRNA genes. **Supplementary Table 2** includes the sequences and amplification efficiencies of the primers. Gene expression was analyzed by qPCR using the StepOnePlus real-time PCR system (Applied Biosystems). Standard cycling conditions were 95 °C for 10 min, 40 cycles of 95 °C for 15 s and 60 °C for 30 s. All reactions were performed with technical duplicates. Relative gene expression levels were calculated using the 18S ribosomal RNA as reference gene following the method described by Pfaffl (42).

### 2.12 Statistical analysis

Statistical analyses were conducted using GraphPad Prism (version 8.0.1). Data were tested for normality using the Shapiro-Wilk test and for homogeneity of variances using F-test. Parametric or non-parametric tests were selected accordingly. For comparisons between groups, unpaired two-tailed Student’s t-tests were applied to normally distributed data with homogeneity of variances, while Mann-Whitney tests were used for non-normally distributed datasets or showing heteroscedasticity.

Quantitative PCR data were analyzed using the Pfaffl method (42).

For transcriptomic analyses, differential gene expression was calculated using DESeq2 (43) with Benjamini-Hochberg correction to control for false discovery rate (FDR). Genes with an adjusted p-value (FDR) < 0.05 and |log2 fold change| ≥ 1 (**Supplementary Table 3**).

## 3 Results

### 3.1 Characterization of the transfer capacity of bivalve ISCs

**Figure 1:**
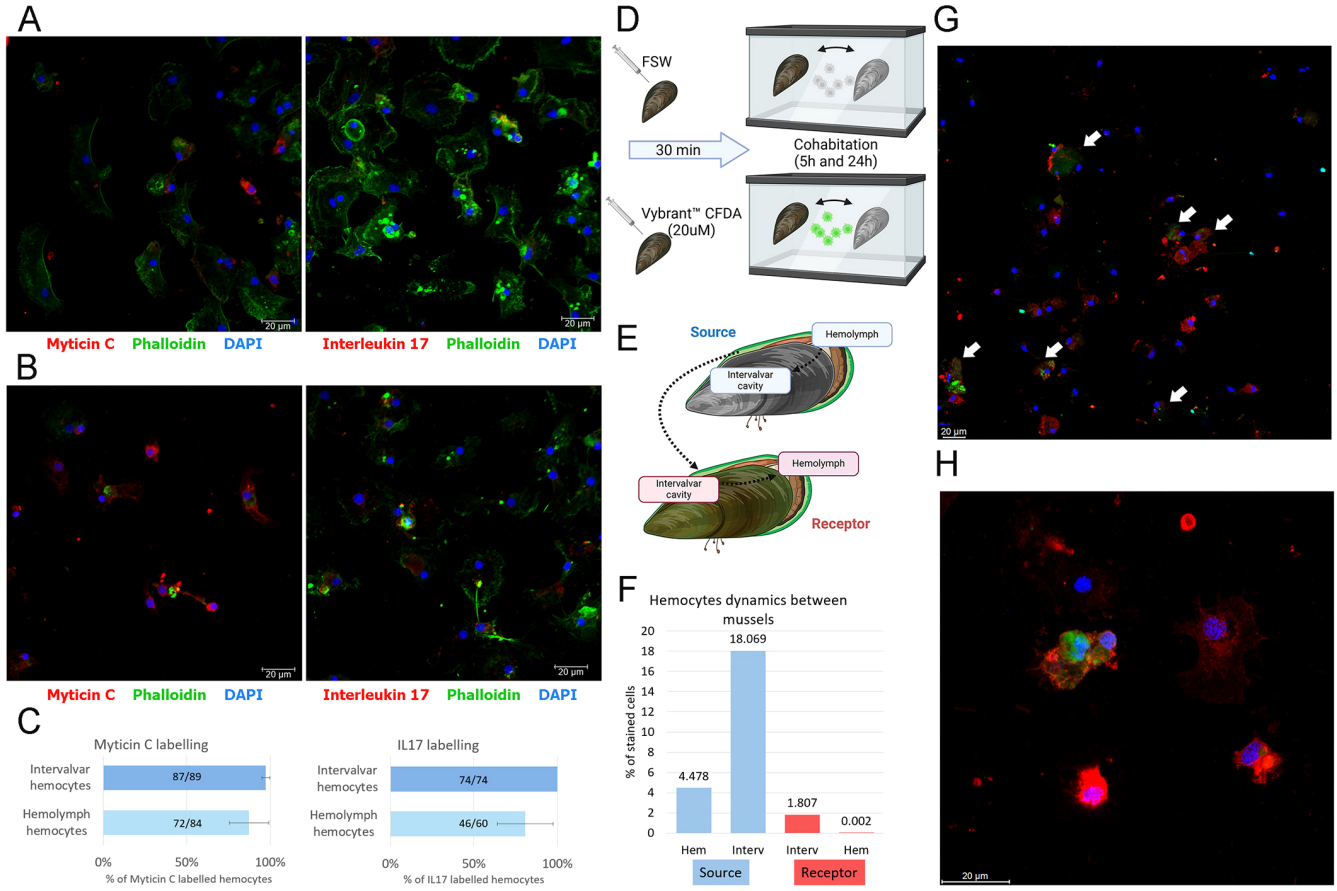
Characterization of ISCs. **(A)** Immunofluorescence of hemolymph hemocytes and **(B)** immunofluorescence of intervalvar hemocytes. Staining was performed using myticin C antibody or interleukin-17 antibody (red), actin with phalloidin (green) and nuclei with DAPI (4′,6-Diamidino-2-phenylindole; blue). Photos were taken at 40x magnification. **(C)** Bar graph showing the percentage of immunostained hemocytes in intervalvar and hemolymph hemocytes. The standard error (± SD) is represented. These differences were not statistically significant (p value=0.5 for myticin C and p value=0.4 for IL17). **(D)** Experimental procedure to assess the hemocytes movements between different individuals. Hemocyte staining was done by injecting Vybrant™ CFDA (carboxyfluorescein diacetate) (20 µM) reagent into the adductor muscle. **(E)** Graph showing the hemocytes movement between two mussels. **(F)** Percentage of stained hemocytes detected in each of the tested animals. These values were obtained by assessing about 160,000 in each case. **(G)** Photo showing cells in the donor/source mussel intervalvar cavity. Green hemocytes incorporated the Vybrant™ CFDA reagent. These cells were also immunostained with myticin C antibody (red). Nuclei were stained with DAPI (blue). The photo was taken at 20x magnification. White arrows highlight hemocytes stained at the adductor muscle of the donor animal that move to the intervalvar cavity of the same animal. **(H)** Photo showing transferred cells in the receptor mussel intervalvar cavity. Colors are the same as in panel **(D)** Photo was taken at 63x magnification.

Hemocytes were immunostained using a myticin C antibody (an antimicrobial peptide specific to *Mytilus* species that is highly expressed in hemocytes) and an interleukin-17 antibody (a highly diverse cytokine implicated in mucosal immunity in mussels). Both antibodies are specific for mussels, providing strong evidence that intervalvar hemocytes are indeed mussel-derived cells functioning outside the organism. The two groups of cells exhibited notable morphological differences in terms of size, cytoplasmic structure and overall morphology (**Figures 1A, B**). Moreover, the percentage of myticin C+ and IL17+ cells was higher in the external hemocytes (**Figure 1C**). Although this result was not statistically significant (p value=0.5 for myticin C and p value=0.4 for IL17), this trend may indicate a primed or active immune status, consistent with their role as frontline responders to microbial challenges.

To determine if this externalization of immune cells is a widespread adaptive trait among bivalves facing similar ecological pressures, we examined the presence of intervalvar cells in other species. Clams and cockles also possess functional immune cells in intervalvar cavities, which confirms the following conserved feature among bivalves: hemocytes of a smaller size than hemolymph hemocytes that can leave the body migrating to the external marine water (**Supplementary Figure 3**).

Having established the presence and potential conservation of intervalvar/external hemocytes across different bivalve species, we next sought to determine whether these cells actively function as part of an externalized immune surveillance system in seawater. This implies active cellular movement, high survival in seawater and even the capacity to reach other individuals. We performed a cohabitation experiment in which mussels shared the same seawater environment following *in vivo* cell staining (**Figure 1D**). Labeled internal hemocytes were tracked by fluorescence and confocal microscopy, and were first detected in the intervalvar space of the source mussel before being released into the surrounding seawater. After that, labeled hemocytes were found in the intervalvar space of the receptor mussel, confirming direct cell transfer between individuals. Finally, stained cells were also found in the internal hemolymph of the receptor mussel (**Figure 1E**). The labeled hemocyte percentage in each location supports these dynamics (**Figure 1F**). A double staining with myticin C antibody enabled the visualization and confirmation of the transfer and specificity of these cells (**Figures 1G, H**). Our findings provide strong evidence that intervalvar hemocytes can move and survive outside the host and suggest the potential for mussel hemocytes to circulate between individuals.

### 3.2 Innate alloimmune response of hemocytes

**Figure 2:**
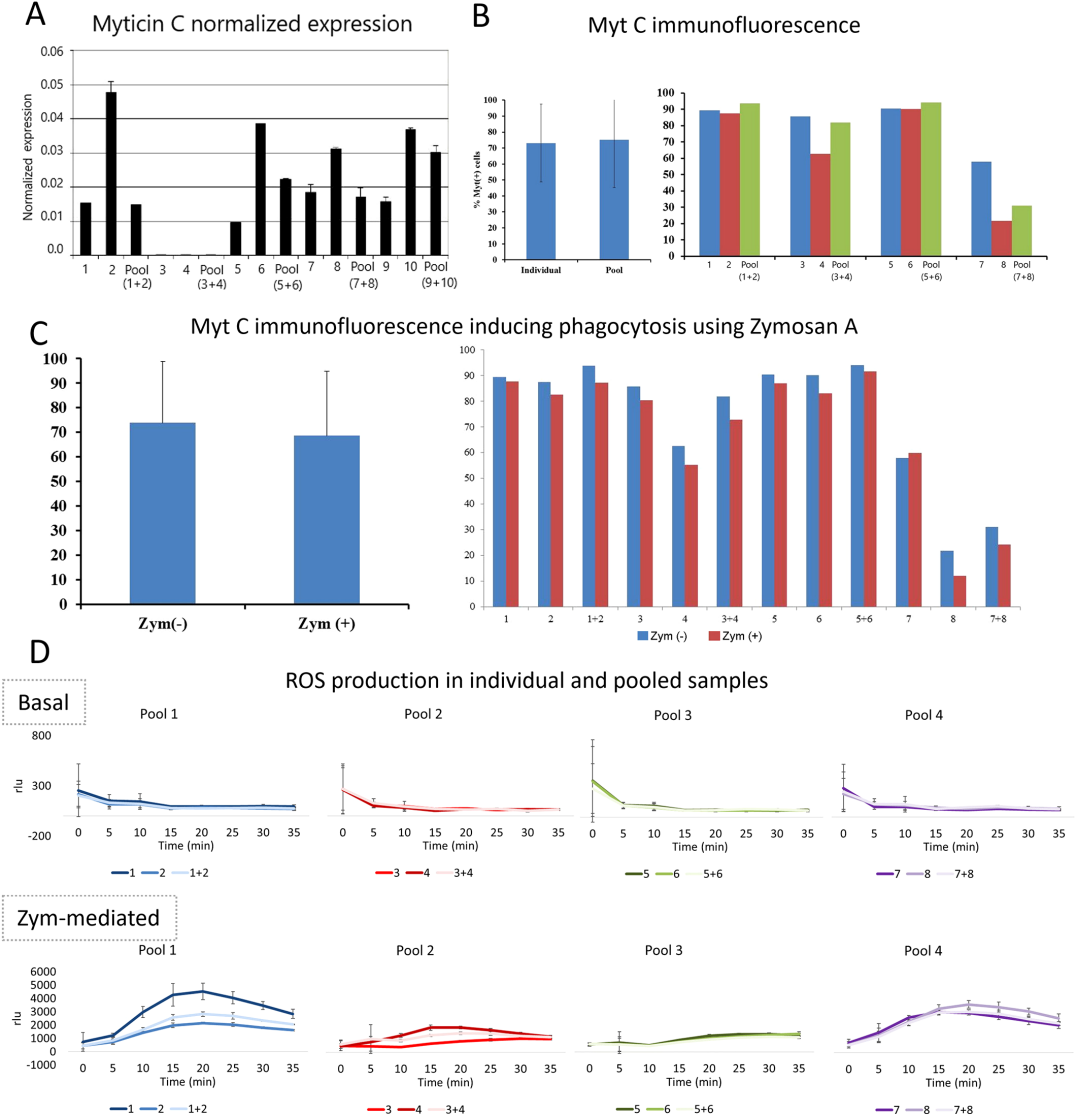
Innate alloimmune response of hemocytes. **(A)** Bar graph showing the myticin C expression after mixing hemocytes of individuals by pairs. **(B)** Myticin C fluorescence after performing an immunofluorescence in hemocytes of individual mussels and mixed hemocytes. The myticin C fluorescence was not statistically significant after comparing hemocytes obtained from individuals and pools (p value=0.5). **(C)** Myticin C immunofluorescence in hemocytes of individual mussels and mixed hemocytes after a phagocytosis activation using zymosan **(A)** These differences were not statistically significant (p value=0.2). **(D)** ROS (Reactive Oxygen Species) production of hemocytes comparing individual and mixed cells. ROS values are represented in rlu (relative luminescence units).

Considering the remarkable movement capacity of bivalve hemocytes and the aforementioned confirmed exchange of hemocytes between mussels, we investigated the possible immune allorecognition of mussel hemocytes. The procedure consisted of mixing hemocytes from different individuals (in pairs) *in vitro* and conducting several immunological assays to determine how the contact between foreign hemocytes could elicit a response. We found no evidence of allorecognition among hemocytes of different individuals (**Figure 2**). Assays measuring myticin C gene expression, myticin C protein levels (under basal and phagocytosis-activated conditions), and reactive oxygen species production showed no signs of immune activation when hemocytes were mixed.

### 3.3 Specific gene expression profiles reveal adaptive specialization of ISCs

The molecular portrait of the external hemocytes (ISCs) was assessed by comparing their transcriptome with that of hemolymph hemocytes and gills (immune barrier). The differences in gene expression among the three types of samples under naïve conditions were evident (**Supplementary Figure 4**), and reflected their divergent functions. Intervalvar hemocytes exhibited higher expression heterogeneity than the other two samples (**Supplementary Figure 4**), and the transcriptomic profile of gills was characterized by immune and inflammatory responses [all the details can be consulted in **Supplementary Figure 5** and also in a previous study (30)].

**Figure 3:**
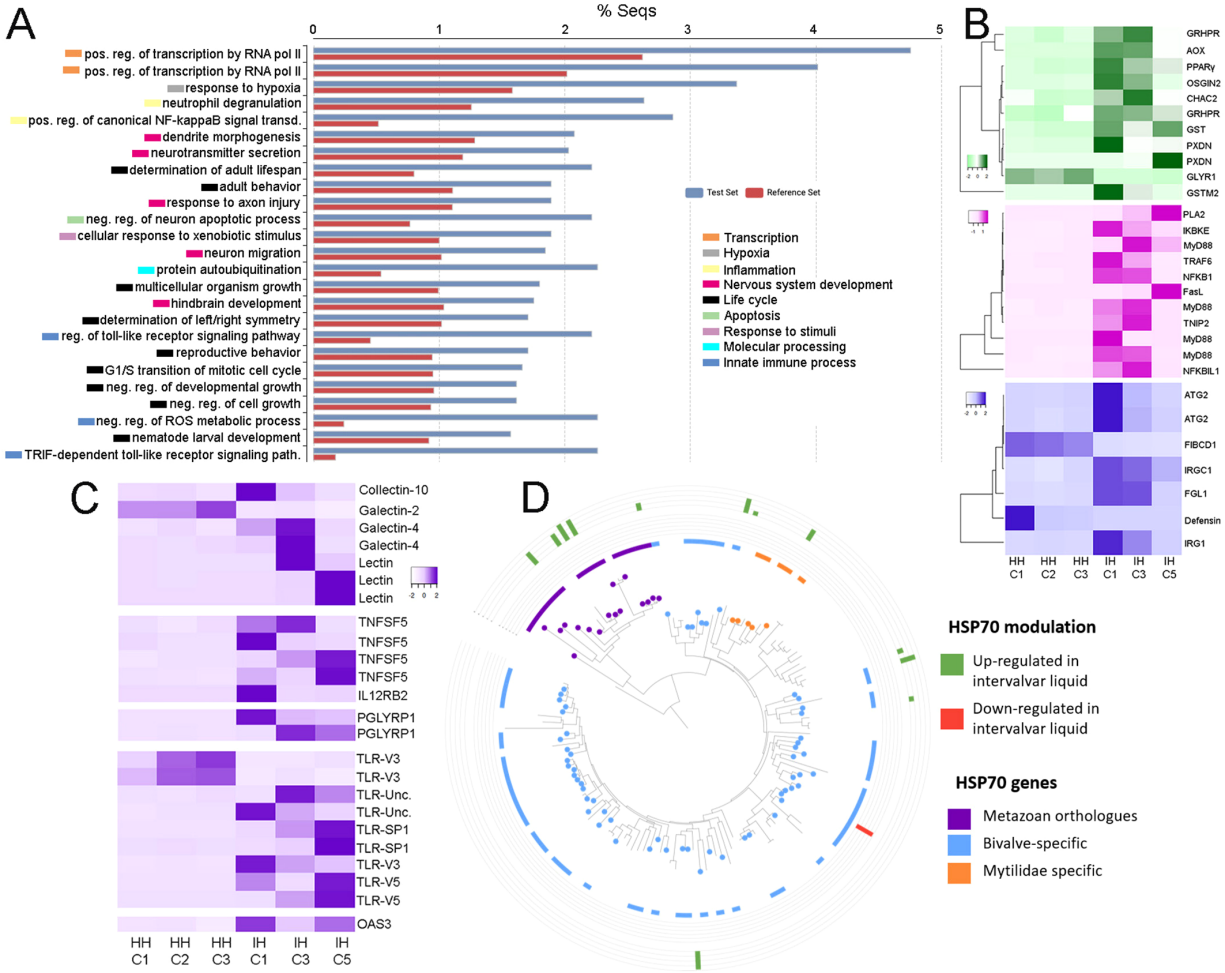
Differential expression between intervalvar hemocytes (ISCs) and hemolymph hemocytes. **(A)** Enrichment analysis showing the main biological processes modulated in ISCs compared to hemolymph hemocytes. **(B)** Gene modulation of oxygen stress-related genes, inflammatory genes and immune-related genes. Heatmaps display TPM values for the two hemocyte groups: intervalvar (IH) and hemolymph hemocytes (HH). **(C)** Gene modulation of pattern recognition receptors. Heatmap shows TPM values for the two hemocyte groups: intervalvar (IH) and hemolymph hemocytes (HH). **(D)** HSP70 modulation in ISCs compared to hemolymph hemocytes. Phylogenetic graph shows the orthology of the gene.

The transcriptomic contrast between intervalvar/external hemocytes (ISCs) and hemolymph hemocytes was of utmost importance, as it helped to define the specific characteristics of these extracorporeal cells. The modulated genes (2,166 DEGs) were generally associated with transcription, cell division, response to stimuli, apoptosis and innate immune response (**Figure 3A**). Intervalvar hemocytes showed an over-expression of transcripts related to oxidative stress regulation, inflammation processes and genes involved in immune responses (**Figure 3B**). Moreover, the expression activation of a significant group of membrane-associated receptors was observed (**Figure 3C**). Among these receptors were several lectins, tumor necrosis factor receptors (TNFSF5), Peptidoglycan recognition protein 1 (PGLYRP1) and several toll-like receptors (TLRs V3, V5 and SP1) (55). The important regulation of these pattern recognition receptors in ISCs suggests an enhanced state of readiness and an adaptation to their role in sensing threats in the microbially rich intervalvar space. In addition, HSP70 genes, related to the animals’ response to environmental stresses (thermal stress, salinity or hypoxia), were among the most expressed in intervalvar hemocytes. HSP70 genes are highly expanded in bivalves, which present both ortholog genes to the HSP70s of other metazoans and also specific genes with no orthology conservation outside bivalves (**Supplementary Figure 6**). As it can be seen in the phylogenetic analysis of mussel’s HSP70s (**Figure 3D**), only a few of these genes, concentrated in a single branch, are orthologs to the metazoan HSP70 family. Strikingly, the rest of the HSP70s were bivalve or even mussel specific in terms of orthology relations, and they account for the majority of the phylogenetic tree, indicating that these specific genes are very diverse in comparison to metazoan ones. This expansion and diversification represent a key evolutionary feature of bivalves. HSP70 genes were over-expressed in intervalvar hemocytes with respect to hemolymph hemocytes (both the metazoan orthologs and the bivalve specific genes), which marks a distinctive feature of these external cells (**Figure 3D**).

### 3.4 Apoptosis resistance: A key adaptation of ISCs for extracorporeal survival

**Figure 4:**
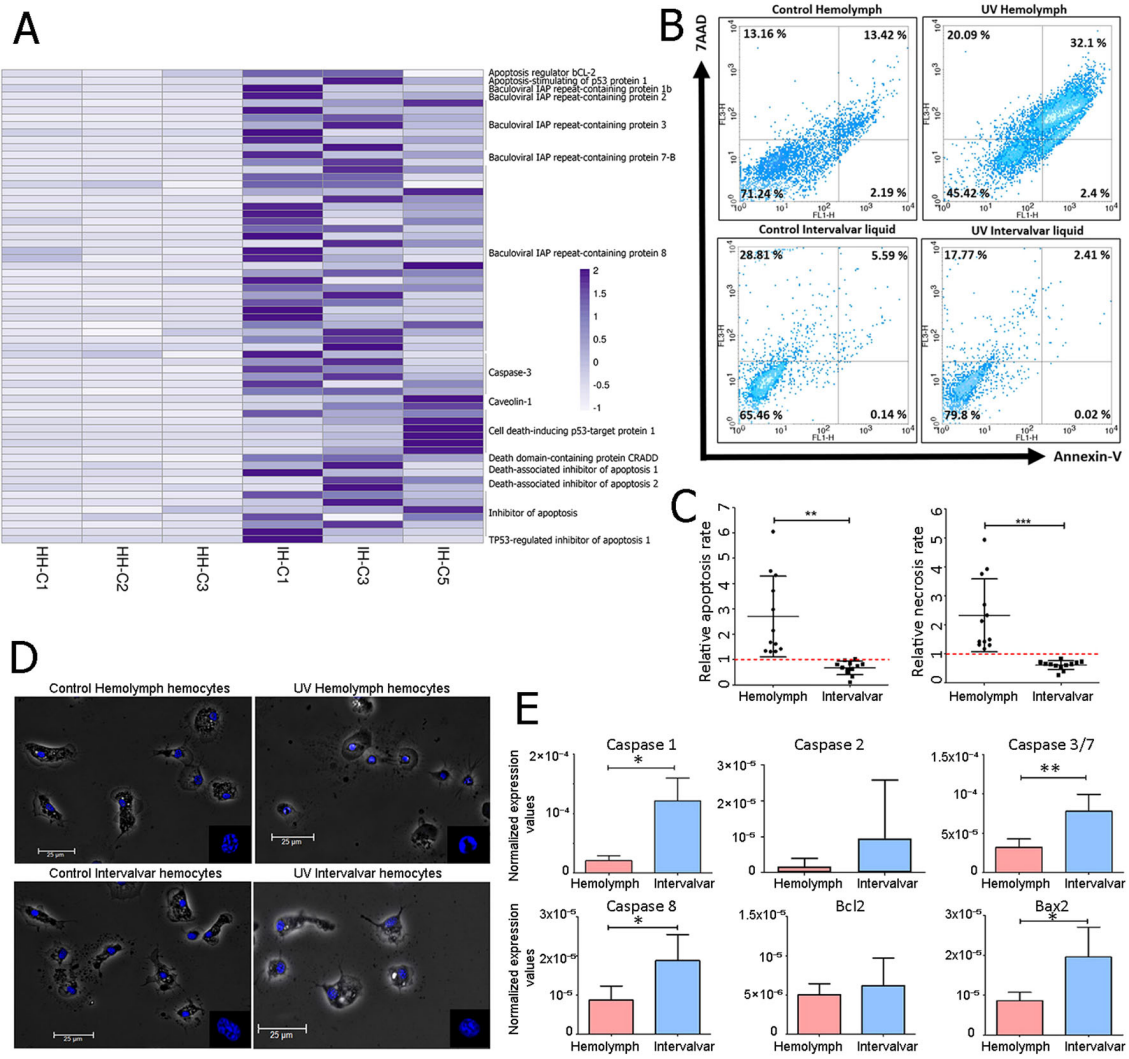
Apoptosis inhibition of ISCs. **(A)** Genes related to apoptotic processes differentially expressed between intervalvar hemocytes and hemolymph hemocytes. The heatmap shows the TPM values of hemolymph hemocytes (HH) and intervalvar hemocytes (IH). **(B)** Annexin V/7AAD (7-Aminoactinomycin D) Flow cytometry analysis of intervalvar hemocytes and hemolymph hemocytes. The Y-axis indicates hemocytes stained with 7AAD and the X-axis indicates hemocytes stained with Annexin V. Viable, early apoptotic, late apoptotic and necrotic hemocytes are represented by the lower left (Annexin V -/7AAD -) lower right (Annexin V +/7AAD -), upper right (Annexin V +/7AAD +) and upper left (Annexin V -/7AAD +) quadrants, respectively. The average percentage of all samples is shown in each quadrant. **(C)** The apoptotic ratio was obtained by dividing the percentage of apoptotic (left) and necrotic (right) cells between the UV-treated sample and control sample in hemolymph and intervalvar samples. **(D)** Phase contrast image showing control and UV-treated hemocytes from hemolymph and intervalvar liquid. Nuclei are stained in blue with DAPI (4′,6-Diamidino-2-phenylindole). The scale bar was set at 25 μm. **(E)** qPCR normalized expression of selected apoptotic genes in hemolymph hemocytes and intervalvar hemocytes. Results represent the average value ± SD (5 biological replicates). Asterisks indicate significant differences at p<0.05 (*) and p < 0.01 (**).

RNA-seq results revealed a notable difference in apoptosis regulation between intervalvar hemocytes and hemolymph hemocytes. **Figure 4A** shows the transcriptomic activation in intervalvar hemocytes of both activators (caspase-3 or death domain-containing protein) and inhibitors of apoptosis (death-associated inhibitor of apoptosis 1 or baculoviral IAP repeat-containing proteins). This complex regulation suggested a finely tuned control over programmed cell death, prompting further investigation into the cells’ functional response to apoptotic stimuli. Therefore, we conducted an Annexin/7AAD analysis by flow cytometry comparing apoptotic processes in hemolymph and intervalvar hemocytes. Early and late apoptosis in hemolymph hemocytes was slightly higher than in intervalvar hemocytes (**Figure 4B**). After using UV light as an apoptosis inducer, the number of early and late apoptotic cells was notably higher in hemolymph hemocytes than in intervalvar hemocytes (2.4% vs. 0.02% and 32.1% vs. 2.41%, respectively) (**Figure 4B**). These differences between both cellular groups were statistically significant when comparing apoptosis and necrosis rates in UV-stimulated and control samples. The absence of response of hemocytes present in intervalvar space is striking (**Figure 4C**).

Moreover, the induction of apoptosis using UV light produced a rounder shape and fragmented chromatin in hemolymph hemocytes. In contrast, intervalvar hemocytes treated with UV light did not show such changes in morphology, neither in the shape of the whole cells nor in the nuclei (**Figure 4D**).

A qPCR gene expression analysis validated the differences described so far between the two groups of hemocytes. Several activators and inhibitors of apoptosis were selected for this purpose (caspase 1, caspase 2, caspase 3/7, caspase 8, Bcl2 and Bax2). Caspase 1, 3/7, 8 and Bax2 were significantly up-modulated in intervalvar hemocytes (**Figure 4E**). Taken together, these data suggest that intervalvar hemocytes maintain a tightly regulated apoptotic program, favoring survival under environmental stress.

### 3.5 Transcriptomics of intervalvar and MtrBTN hemocytes reveals adaptations for external survival

Given that transmissible cancers - fatal leukemia-like neoplasias - have been documented in bivalves (37, 38), we considered the possibility that neoplastic hemocytes and intervalvar hemocytes might share certain gene expression traits that enable them to persist in seawater and successfully enter new hosts. To evaluate this, we conducted an analysis comparing the expression profile of external hemocytes with those of neoplastic and control hemocytes from *Mytilus edulis* (MtrBTN; PRJNA749800 (44)). All the samples were mapped to the same *M. galloprovincialis* genome reference used in the rest of the analyses of this work, yielding equivalent mapping rates between the two mussel species.

**Figure 5:**
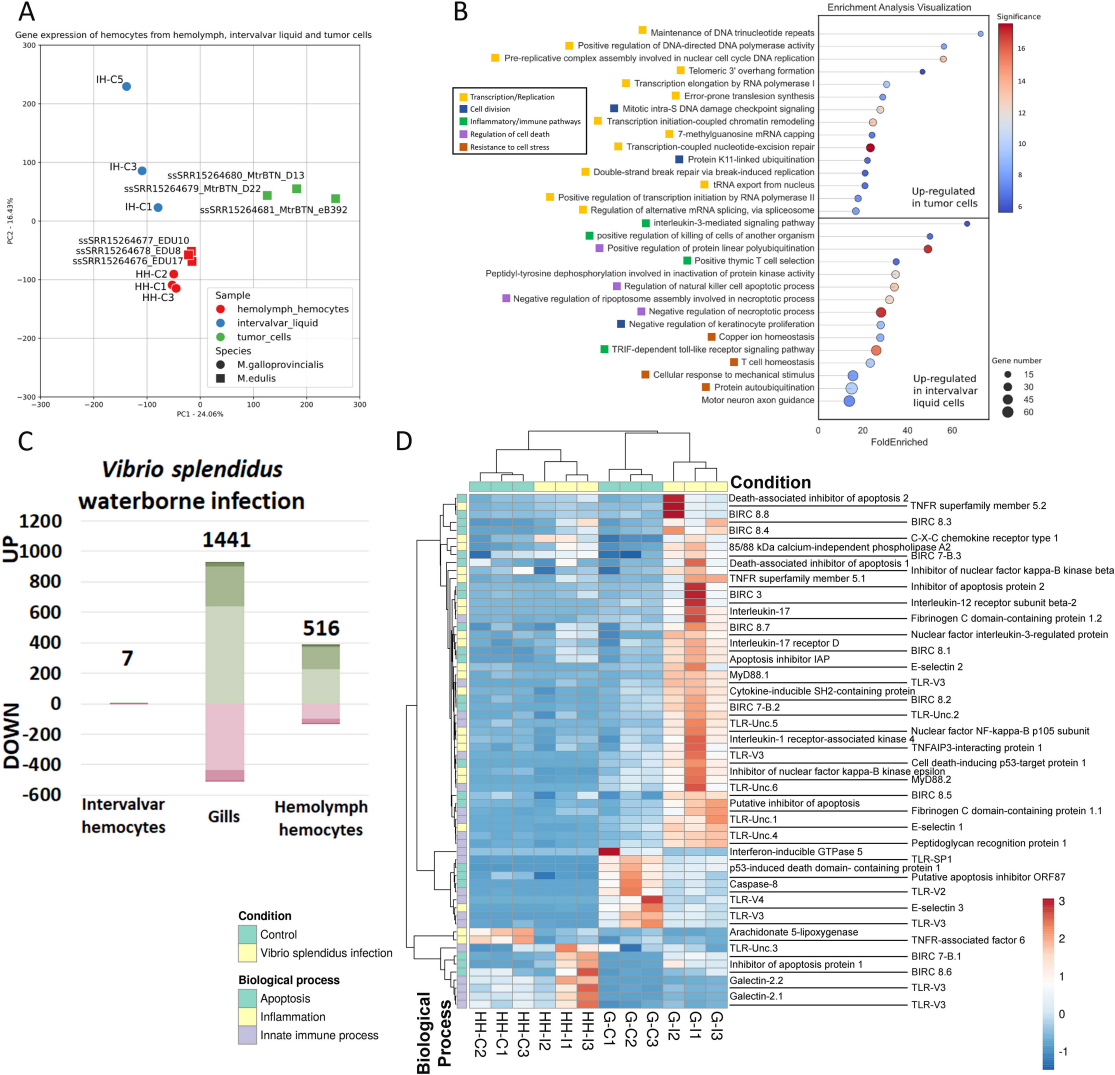
Transcriptomic profile of intervalvar hemocytes (ISCs) compared to MtrBTN hemocytes and in an infection context. **(A)** PCA displaying the total transcriptomic profile of hemolymph hemocytes, intervalvar hemocytes and MtrBTN hemocytes. The control hemolymph hemocytes and intervalvar hemocytes from the current work were used, in addition to *M. edulis* hemolymph hemocytes and MtrBTN hemocytes from previous work (PRJNA749800). All samples were mapped to the same *M. galloprovincialis* reference used in the rest of the analyses of this work with equivalent mapping rates. **(B)** Enriched biological processes after the transcriptome comparison of intervalvar hemocytes and MtrBTN hemocytes. This panel shows the difference in expression between the two cell groups. **(C)** Bar graph showing the number of differentially expressed genes in intervalvar hemocytes, gills and hemolymph hemocytes (Log2FC>|1|; adjusted p-value<0.05) after a bacterial waterborne infection. Note the absence of modulation in intervalvar hemocytes. **(D)** Heatmap showing the modulation of the genes involved in the bacterial response. A wide range of genes involved in apoptosis, inflammation and general innate immune recognition processes are up-modulated in gills after the infection. Hemocytes exhibit minimal immune modulation after a waterborne infection.

The total transcriptomic profile of hemolymph hemocytes, external ISCs and MtrBTN hemocytes is shown in **Figure 5A** and **Supplementary Figure 7A**. There was an evident difference in general expression between hemolymph hemocytes and the other samples. Neoplastic hemocytes and external ISCs shared expression features related to cellular and cilium motility, as well as resistance to stress such as exposure to seawater (“protein refolding”, “response to redox state”, “homeostasis of number of cells within a tissue”) (**Supplementary Figure 7B**).

Although these two samples (ISCs and neoplastic hemocytes) share a range of similarities, they also present evident differences since the defining transcriptomic profile of neoplastic hemocytes was enriched in genes related to cell replication evidenced by the biological processes related to cell cycle, replication and transcription (**Figure 5B**). In contrast, the defining transcriptomic profile of ISCs was enriched in genes related to stress resistance, homeostasis, osmotic stress, and chaperone-related, which are linked to the exposure to seawater, but also presented a marked inflammatory and immune profile, processes which are inhibited in the mussel neoplastic cells (**Figure 5B**).

### 3.6 ISC tolerance to acute bacterial challenges

In addition to the inherent ISCs characteristics studied in this work, the response to a waterborne infection using *Vibrio* sp*lendidus* was also analyzed. Strikingly, external hemocytes, the first cells to encounter the pathogen showed no transcriptomic response to the infection (**Figure 5C**). This lack of response is a key finding and may be a consequence of an evolved tolerance to continuous contact with microorganisms in the intervalvar cavity. In fact, in gills, the next barrier facing the challenge as well as in hemolymph hemocytes, the response was evident (**Figure 5C**). In both cases, gills and hemolymph hemocytes, the response was driven by stimulus-response genes and also inflammatory processes (**Figure 5D**; **Supplementary Figures 8A, B**). A broad range of genes involved in pathogen recognition, such as toll-like receptors or peptidoglycan recognition proteins, showed significantly increased expression levels only in the gills after the bacterial waterborne infection. This also happened with inflammatory genes such as tumor necrosis factor receptors, interleukin-17 and its receptors, Myd88 or nuclear factor KF-kappa-B (**Figure 5D**). This robust response in the gills highlights their role as a primary internal defense barrier, in contrast to the tolerant phenotype of the external ISCs.

### 3.7 Microbial communities inhabiting the intervalvar space: the ecological context of mussel ISCs

**Figure 6:**
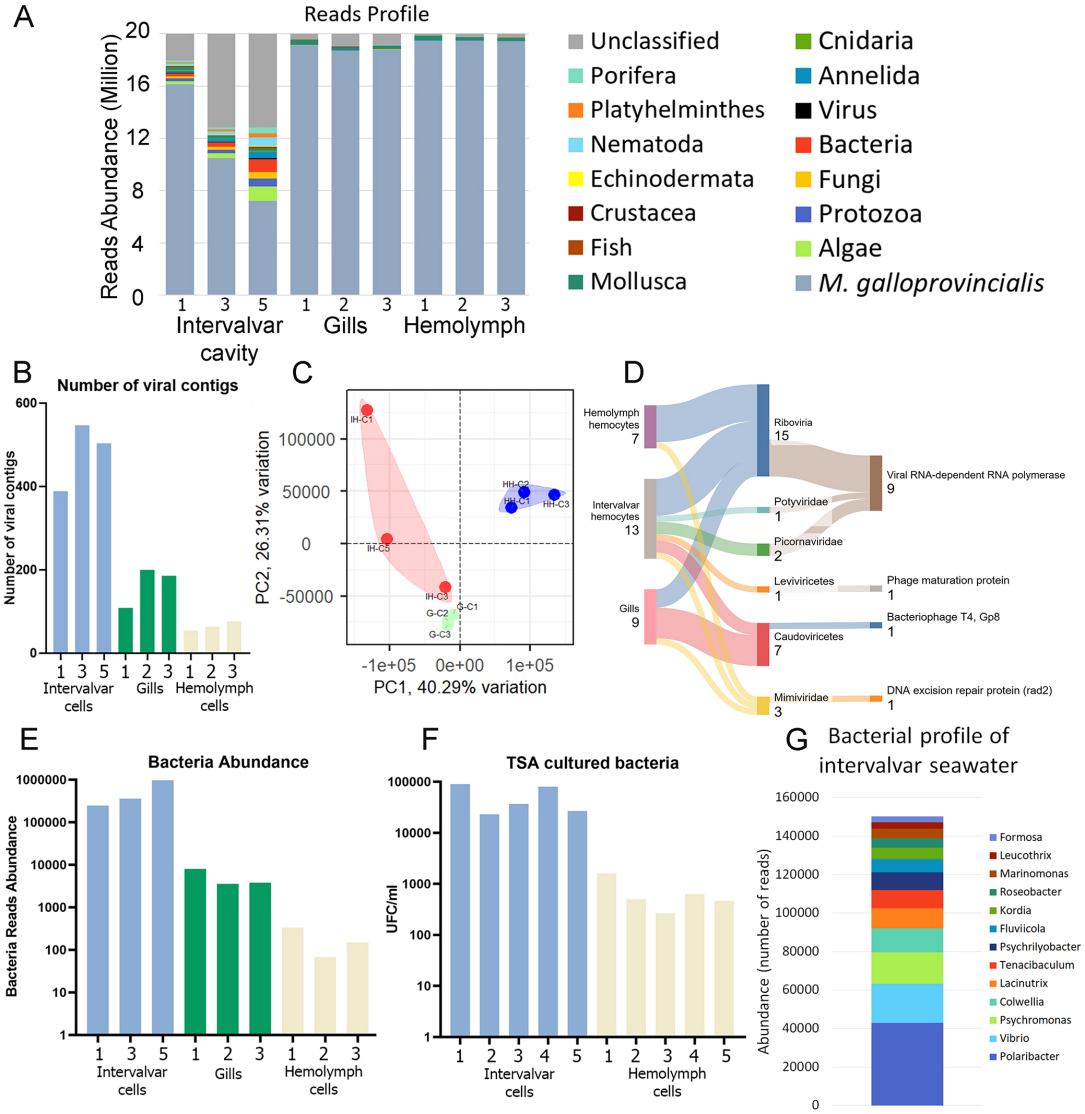
Microbiome definition of *M. galloprovincialis* ISCs. **(A)** Taxonomic classification of reads from intervalvar seawater, hemolymph and gills. **(B)** Number of viral contigs identified in naïve mussels. **(C)** Principal components analysis showing the differences in viral composition among sample types [intervalvar hemocytes (IH), hemolymph hemocytes (HH) and gills (G)]. **(D)** Taxonomic classification of viral contigs detected in intervalvar seawater, hemolymph and gills. This figure also shows the detected domains associated with each taxon. **(E)** Number of reads classified as bacteria in naïve mussels. **(F)** The number of bacterial colony-forming units (CFU/mL) was counted in a validation experiment using intervalvar liquid and hemolymph. **(G)** Bacterial profile associated with ISCs and identified in the intervalvar cavity.

Since the intervalvar cavity is where interactions between the host immune system and the external environment primarily occur, we performed analyses to define its microbial composition. Trimmed reads were mapped to the *M. galloprovincialis* genome (27), and around 30-70% (depending on the sample) of reads retrieved from intervalvar seawater did not map to the mussel genome (**Supplementary Table 4**; **Figure 6A**). These unmapped reads were classified using different databases, including prokaryote and several eukaryote genomes. Intervalvar seawater samples exhibited a substantially larger non-mussel fraction, composed of reads from diverse microorganisms and eukaryotes.

The virome of the intervalvar seawater, gills and hemolymph was evaluated for comparative purposes (**Supplementary Figure 2**). A total of 3,193 viral contigs were found. Intervalvar seawater contained more viral contigs than gills and hemolymph (**Figure 6B**), and the composition was also more diverse (**Figure 6C**). In some cases, the taxonomic classification of viral contigs was possible at the family level (**Figure 6D**). Two Picornaviridae and one Potyviridae were exclusively found in intervalvar samples. Viral contigs belonging to the Mimiviridae family were also found inside the mussel bodies. Some other taxonomic classifications were possible, but at higher levels (Leviviricetes and Caudoviricetes).

In the same direction, bacterial load (**Figure 6E**) was 10-fold more abundant in intervalvar seawater than in gills and 100-fold more than in hemolymph. To validate this result, hemolymph and intervalvar seawater samples were seeded on TSA plates, and colony-forming units (CFUs) were counted after 24 h. The viable bacteria counts were 10 to 100-fold higher in intervalvar seawater than in hemolymph (**Figure 6F**), confirming the trend demonstrated by transcriptomic reads. Regarding the bacteriome composition, we could classify about 500 thousand reads on average in intervalvar seawater samples. The most abundant genera were *Polaribacter*, *Vibrio* and *Psychromonas* (**Figure 6G**).

## 4 Discussion

The capacity of organisms to adapt to microbially rich environments is a cornerstone of evolutionary success. Our findings suggest the presence of an externalized immune sentinel system composed of specialized hemocytes (ISCs) that might operate outside the conventional body limits in marine bivalves. This discovery expands our understanding of how marine metazoans, particularly invertebrates, have evolved mechanisms to defend against complex environments (22, 23).

Several marine metazoan phyla, including bivalves, feed by filtering seawater, which implies that they internalize organisms in suspension. Despite this high exposure to putative pathogenic microorganisms, toxins and contaminants, they have entirely adapted by adopting different strategies to overcome these challenges. This is true for mussels. The sequencing of the *Mytilus galloprovincialis* mussel genome (27) revealed the first open pangenome in a metazoan, with a large fraction of dispensable genes related to its adaptation capacity (56). In addition, this dispensable fraction is enriched in immune gene families, many of which are highly expanded and specialized at the functional level (40, 55). Recent reports suggest that pangenomes and immune gene expansions may be common evolutionary adaptations among most bivalves (57). Our work builds upon this understanding suggesting a cellular-level adaptation that likely complements these genomic features. Intervalvar hemocytes are active immune sentinel cells (ISCs) that would participate in external immune networks.

We visualized the ability of internal hemocytes to exit the body, reach the seawater, and get into another mussel individual. This inter-individual cellular exchange, occurring via a continuous flow of hemocytes from the adductor muscle to the intervalvar cavity and then into the external environment before reaching a recipient, has profound implications for understanding population-level immunity as defense strategies in these sessile organisms.

We also characterized major changes that these external hemocytes undergo when they leave the body which define them as ISCs. Their transcriptome showed a strong activation of pattern recognition receptors, including lectins, cytokine receptors and TLRs. This resembles vertebrate sentinel cells, which use these receptors to initiate the immune responses (1, 3). Furthermore, ISCs exhibit the activation of pathways related to the production of reactive oxygen species, general inflammation and physical stress resistance.

Perhaps one of the most surprising adaptations of mussel ISCs is their resistance to apoptosis, even when exposed to potent inducers like UV light. This likely explains their ability to survive in seawater, where they are exposed to natural light. Their capacity to avoid programmed cell death supports their resilience in the environment and the potential to colonize new individuals as immune sentinels. The cellular stress caused by the cellular movement is mitigated by up-regulation of genes such as heat shock proteins and other chaperones in external hemocytes. Notably, HSP70, a suppressor of apoptosis (58), is among the most regulated genes in the transcriptome comparison. This regulation would be related to the capacity of these cells to achieve long-term survival in seawater, as also shown by the comparison of their transcriptomic profiles with those of neoplastic cells. Orthologs of HSP70 genes found in other metazoans would be implicated in the conserved functions ascribed to these genes across animal species, particularly in helping cells cope with environmental stresses like prolonged seawater exposure. Interestingly, several other modulated HSP70 variants show no orthology with known metazoan proteins, suggesting they may represent unique adaptations specific to bivalves and mussels. This aligns with previous findings indicating that gene families exhibiting expansions and high genetic diversity in certain taxa often undergo functional diversification, which supports the metabolic demands of maintaining such genomic complexity (55, 57).

The transfer of cells between individuals could be related to the mechanism of pathology transmission, including the disseminated neoplasia, a leukemia-like cancer of bivalves, one of the few examples of transmissible cancers. Transmission was thought to happen when cancer cells were released into the water by diseased or dead individuals and taken up by filter-feeding healthy individuals (59–62). Our results suggest that the movement of immune cells among individuals is a natural process that could facilitate the spread of tumoral cells. Our results could also support the ability of hemocytes to act as pathogen carriers between bivalve individuals. Infected hemocytes may enter naive bivalves and cause the dissemination of some bacteria and parasites (33, 35). Several ISCs expression features were shared with *M. edulis* cancerous cells, including the high movement rate, the colonization of new individuals, the resistance to stress and the inhibition of programmed cell death. However, their transcriptome is clearly distinguished from neoplastic hemocytes in the expression of immune genes. We have also exposed several signs of lack of allorecognition, which would enable the exchange of hemocytes between different individuals. There are examples of allorecognition evasion in invertebrates such as cnidarian *Hydractinia symbiolongicarpus*, which form colonies after the molecular binding of identical or nearly identical Allorecognition proteins (Alr1 or Alr2) (63), or the colonial tunicate *Botryllus schlosseri*, in which fusion/rejection reactions are controlled by allelic variations in a series of linked genes (fuhc, fester, uncle fester and BHF) (63–65). It remains unclear whether, or how, these molecular factors interact. As a start point, we show several results that would indicate a certain way of evasion of allorecognition in mussels, that need to be further studied. Although the mechanism and functions that hemocyte transfer between individuals may have remain to be determined, it could suggest a more complex and general immune mechanism in bivalves.

Another feature of ISCs is their apparent immune tolerance in a more than exigent environment. Our results showed a high basal expression level of immune effectors in intervalvar hemocytes, which completely disappears in response to waterborne infections. There was a significant shift in expression. As with internal hemocytes, intervalvar hemocytes may also deploy tolerance mechanisms (13), potentially developing a measured inflammatory response after repeated encounters with pathogens such as *Vibrio* sp*lendidus* (13). This phenomenon may resemble “trained immunity” or represent a unique form of peripheral tolerance, potentially linked to the immunological memory previously documented in invertebrates (66, 67). Indeed, their basal immune activation likely reflects an adaptation to this constant microbial interaction (22, 23). Characterizing the intervalvar microenvironment itself provides crucial ecological context for understanding the functioning of ISCs. This unique niche, previously underexplored, exhibits significantly higher microbial biodiversity and load compared to internal tissues (68, 69). Even considering the potential limitations to identify microorganisms using RNA-seq data (high sequence diversity, limited availability of databases, or low coverage of reads), this information enables to continue establishing the bases for accurately identify and determine interactions of microbiome key taxa which directly influences in host immune system.

In summary, our results reveal a novel immune strategy in bivalves. The deployment of immune sentinel cells (ISCs) that operate externally and can be transferred between individuals to manage microbial exposure has implications for our understanding of invertebrate immunity, as well as for host-pathogen interactions. Mussel ISCs show remarkable differences compared to internal hemocytes: i) basal higher immune gene expression, ii) apoptosis evasion, and iii) lack of any response to acute infections with *Vibrio* sp*lendidus*. The ability of the ISCs to maintain a balance between pathogen detection and immune tolerance highlights a kind of environmental immunology that bridges external and internal immune responses. This work underscores the specialized role of ISCs in bivalves and deepens our understanding of how these organisms adapt their immune strategies to their ecological niches. Further research is necessary to understand its implications for immune surveillance and inter-organismal interactions in invertebrates. Next steps will further characterize these cells, determining whether they represent a cell lineage distinct from hemocytes, whether they are more active at the phagocytic level, or whether their migration between individuals has consequences for the recipient individual.

## Data Availability

The datasets presented in this study can be found in online repositories. The names of the repository/repositories and accession number(s) can be found below: https://www.ncbi.nlm.nih.gov/, PRJNA1222121.
