# Supplementary material for: External immune sentinels in the seawater ecosystem: the specialization of bivalve hemocytes
Source: Front Immunol. 2026 Feb 18;17:1765948. doi: 10.3389/fimmu.2026.1765948 (PMC12956514; doi:10.3389/fimmu.2026.1765948)
Supplement: Supplementary file 1 [file Presentation1.zip › Suppl. Figures, Supp. Tables 1 & 2.docx]

Supplementary Material

**
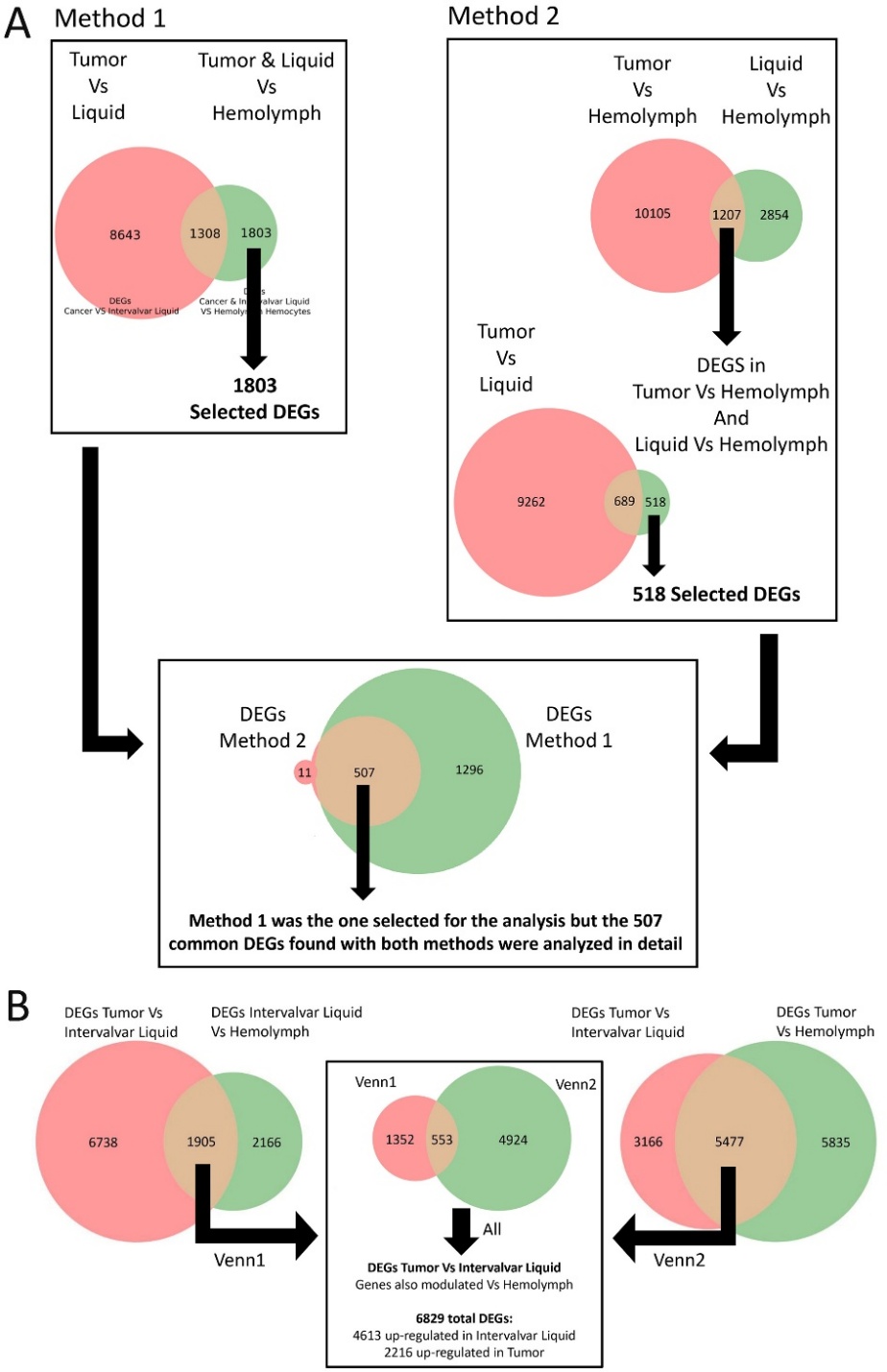
**

Supplementary Figure 1. RNAseq analysis to compare neoplastic hemocytes and ISCs. (A) Two methods to analyze the common transcriptomic profile of hemocytes from intervalvar liquid and mussel neoplastic hemocytes. DEGs shared by intervalvar hemocytes and neoplastic hemocytes were identified by performing a differential expression analysis of these grouped samples against the internal hemocytes samples. DEGs modulated also between neoplastic and intervalvar liquid cells were discarded (Method 1). An alternative method consisted in analyzing separately the modulation of neoplastic and intervalvar hemocytes respectively against internal hemocytes and retrieving the genes modulated in both cases. After that, DEGs also modulated between intervalvar hemocytes, and neoplastic hemocytes were removed (Method 2). The set of genes identified using the alternative method was smaller and included almost entirely within the set of genes found with Method 1, therefore this one was chosen for further analysis. (B) Venn analyses of the transcriptomic data to analyze the differences between neoplastic hemocytes and intervalvar hemocytes.


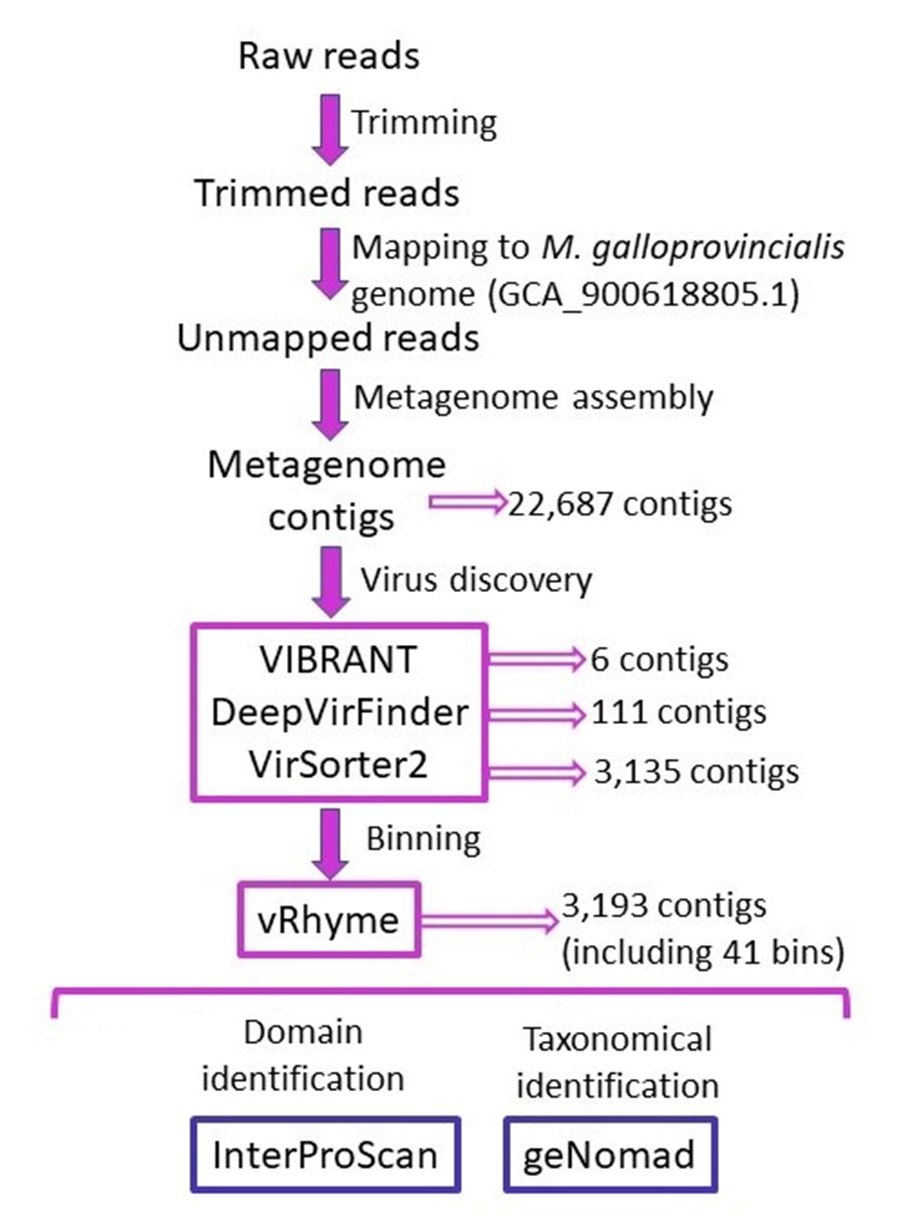


**Supplementary Figure 2.** Work plan to identify viral sequences. Software and steps to analyze transcriptomic data of intervalvar seawater, hemolymph and gills of *Mytilus galloprovincialis*.


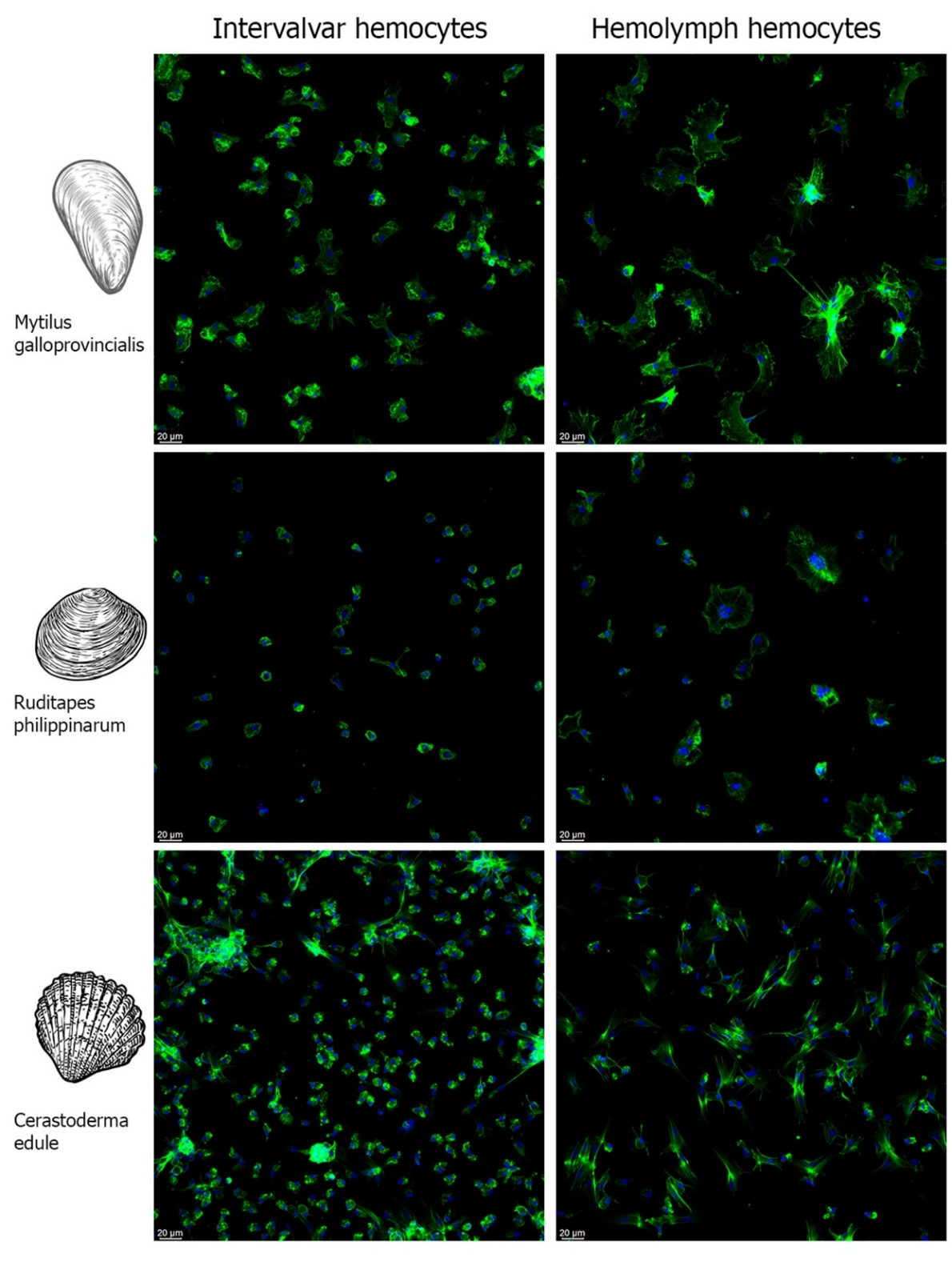


Supplementary Figure 3. Intervalvar and hemolymph hemocytes of bivalves. Fluorescent staining of intervalvar and hemolymph hemocytes of *Mytilus galloprovincialis*, *Ruditapes phillipinarum* and *Cerastoderma edule*. Nuclei are stained with DAPI (4ʹ,6-Diamidino-2-phenylindole; blue), and actin is stained with phalloidin (green).


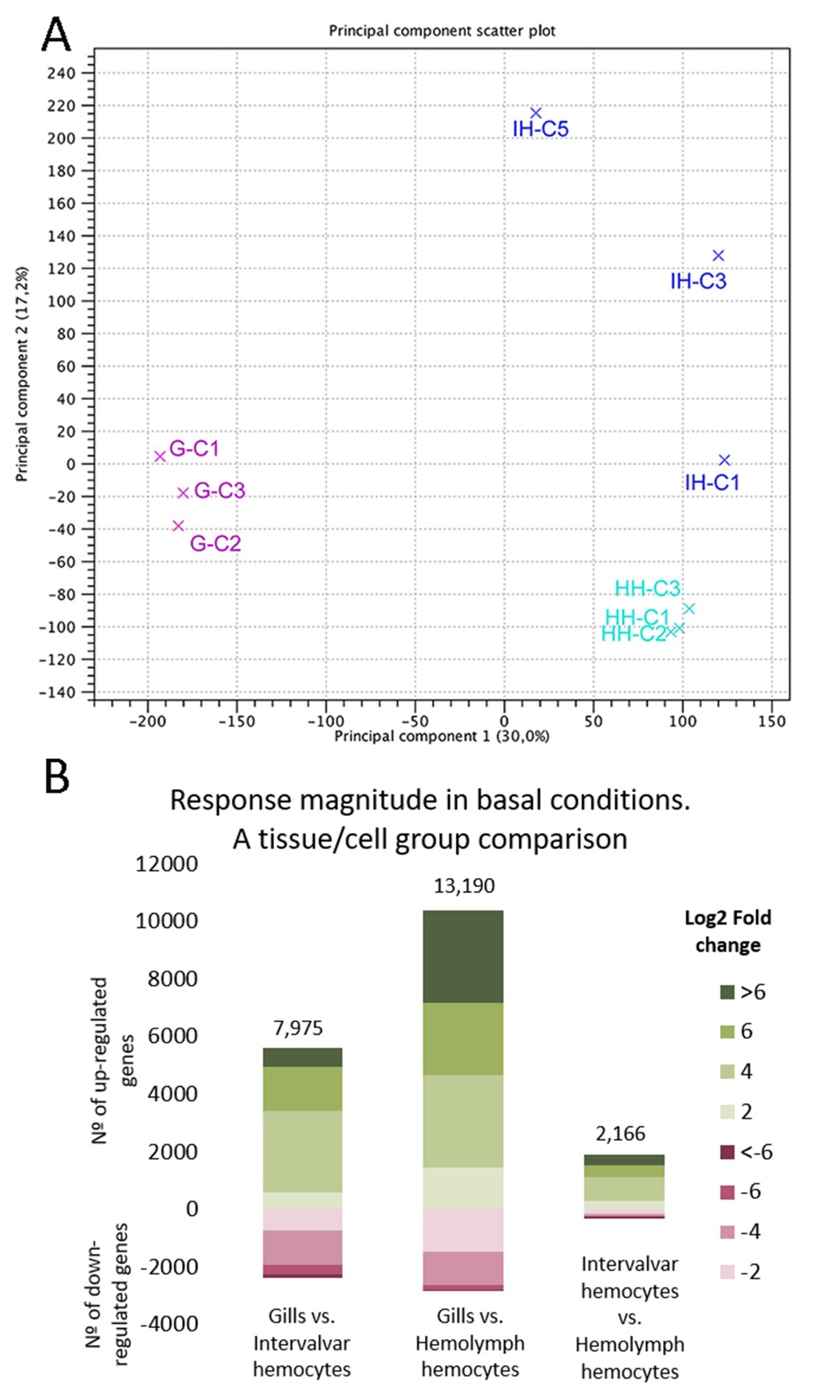


Supplementary Figure 4. General transcriptome expression in all the control samples. (A) Principal components analysis. (B) Gene expression modulation between control samples. Sample IDs are: intervalvar hemocytes (IH), hemolymph hemocytes (HH) and gills (G).


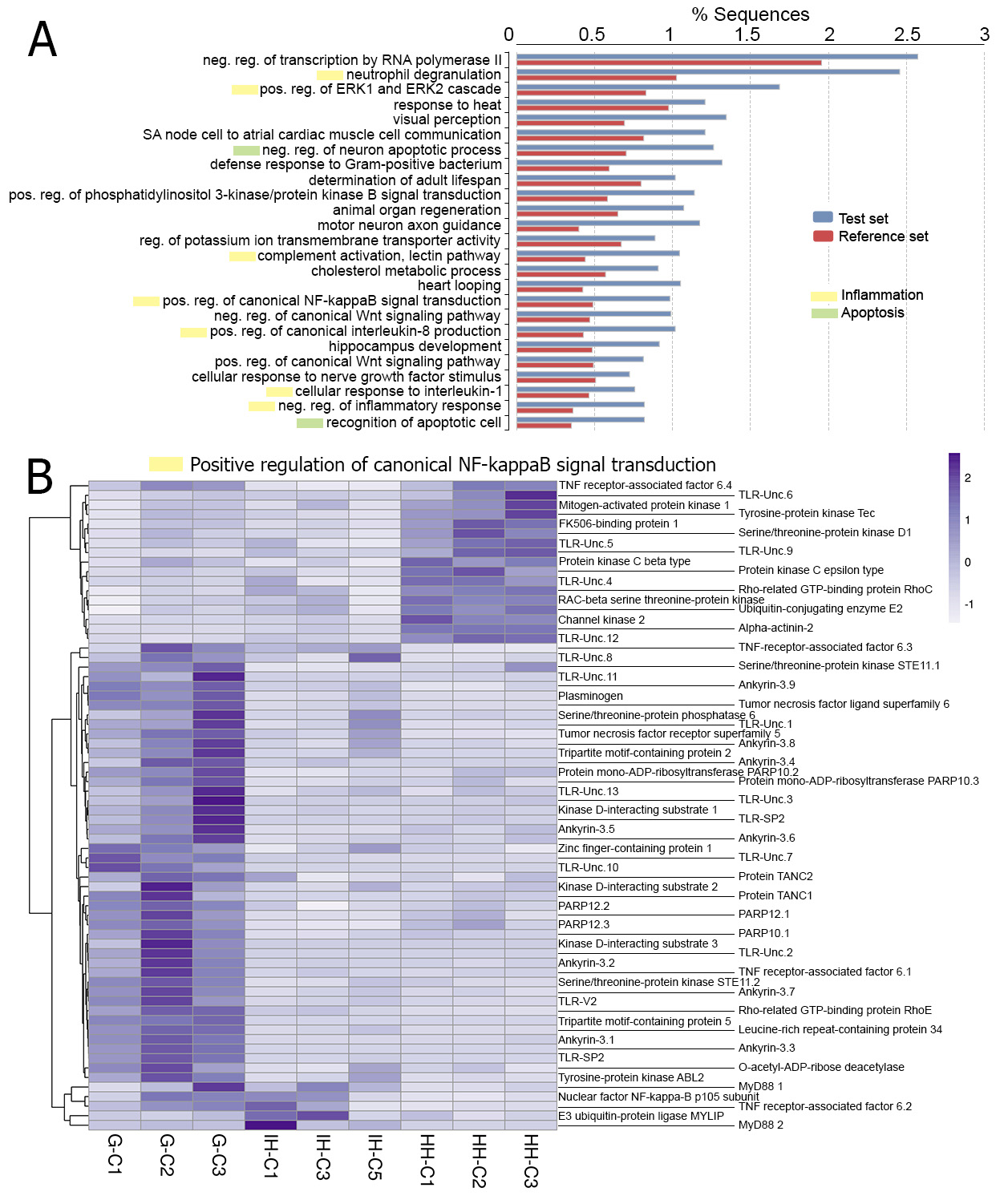


Supplementary Figure 5. General modulation of gills compared to intervalvar hemocytes and hemolymph hemocytes. (A) Enrichment analysis of modulated genes comparing control gills with the two groups of control hemocytes. (B) Heatmap showing the genes activated in NF-kappaB signal transduction. Those genes are mainly activated in gills, followed by moderate activation in hemolymph hemocytes and not expressed in intervalvar hemocytes. The heatmap shows the TPM values of control samples for gills (G), hemolymph hemocytes (HH) and intervalvar hemocytes (IH).


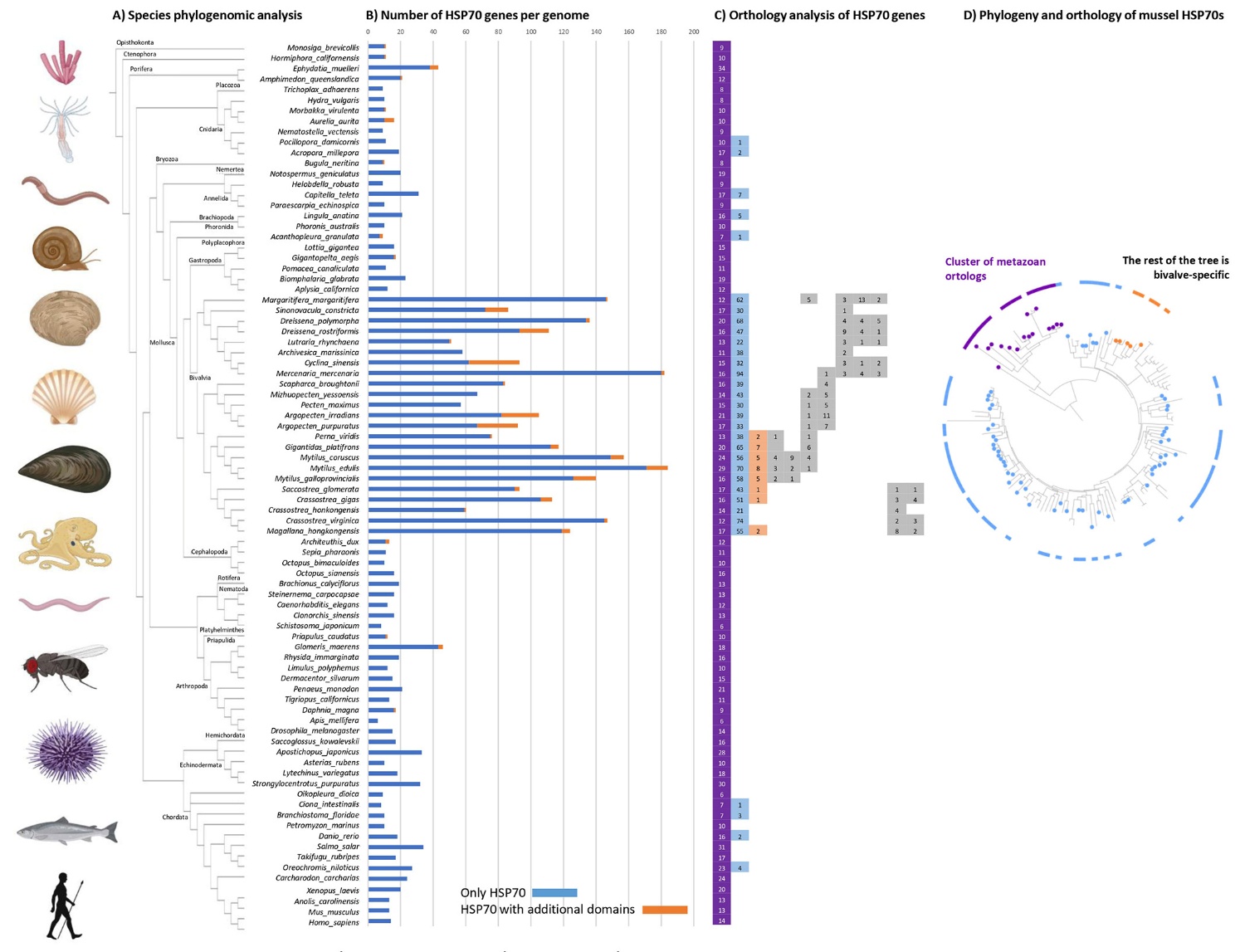


Supplementary Figure 6. Analysis of the HSP70 (Heat Shock Protein 70 gene family). (A) A phylogenomic tree was built with the genomes of the metazoan species that were analyzed in search of their HSP70 repertoires. (B) The identification of HSP70 genes was domain based and we distinguished between genes showing only the HSP70 domain and genes that presented it in combination with other functional domains. A clear expansion in the HSP70 gene repertoire can be seen in all bivalve species. (C) The orthology analyses reported HSP70 orthologs between all species but also genes with orthology restricted to bivalves, which corresponded to the expansions shown before. The number of HSP70 genes in each orthology group is shown. (D) The phylogenetic tree of mussel HSP70s showed that all orthologs to metazoan HSP70 were clustered in a single branch, while the rest of branches corresponded to bivalve or mussel specific genes, which were the vast majority and much more diverse.


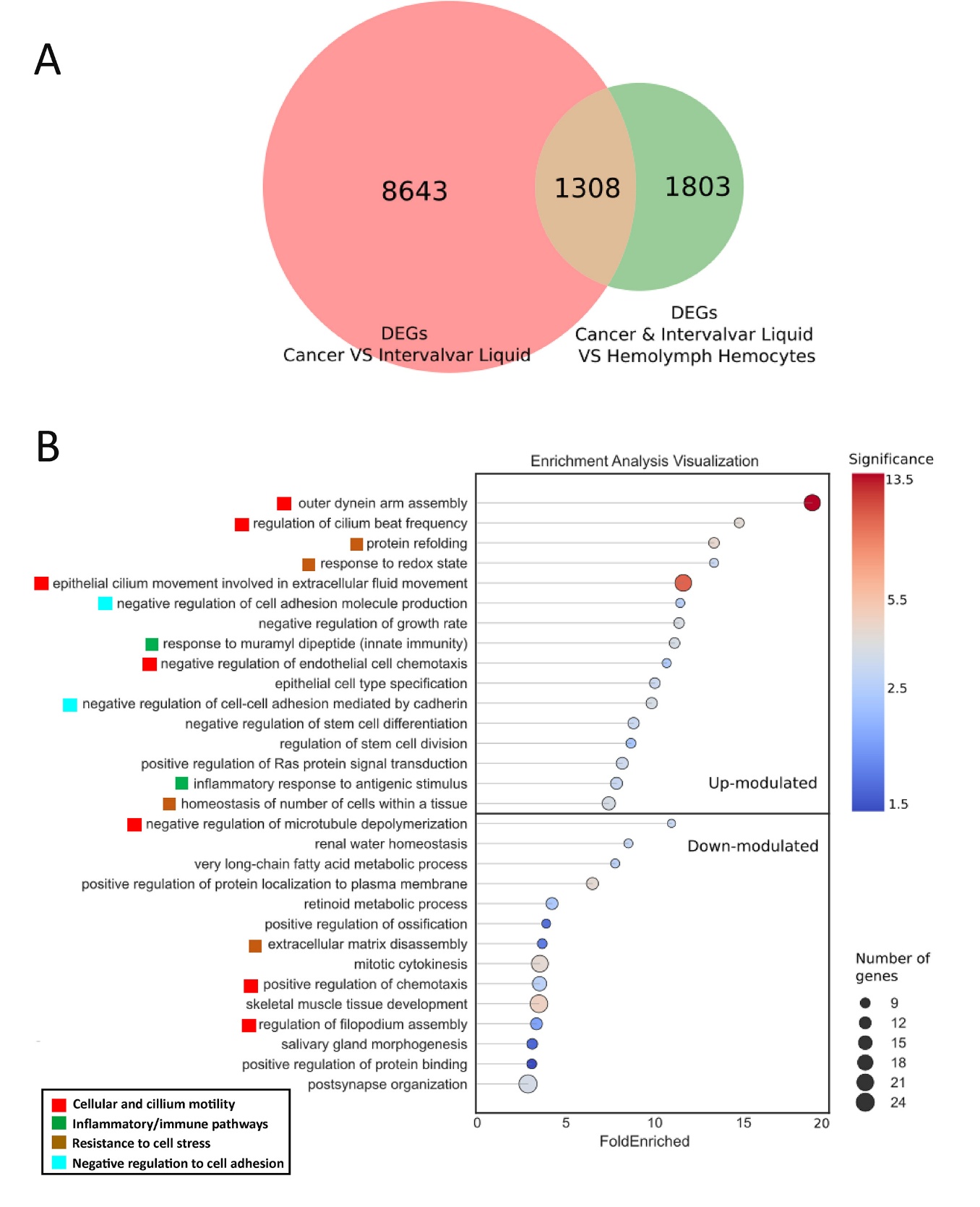


Supplementary Figure 7. Expression comparison between external cells (neoplastic and intervalvar hemocytes) and internal hemocytes. (A) Differentially expressed genes, DEGs (adjusted p-value <0.05 and log2FC>|1|), were obtained between the set of samples of intervalvar hemocytes, neoplastic cells and hemolymph hemocytes samples. (B) Enriched biological processes comparing intervalvar hemocytes and MtrBTN hemocytes to the transcriptome of hemolymph hemocytes. Processes and their “Fold Enriched” rate between the test and reference sets are represented. Their significance and number of genes are also shown.


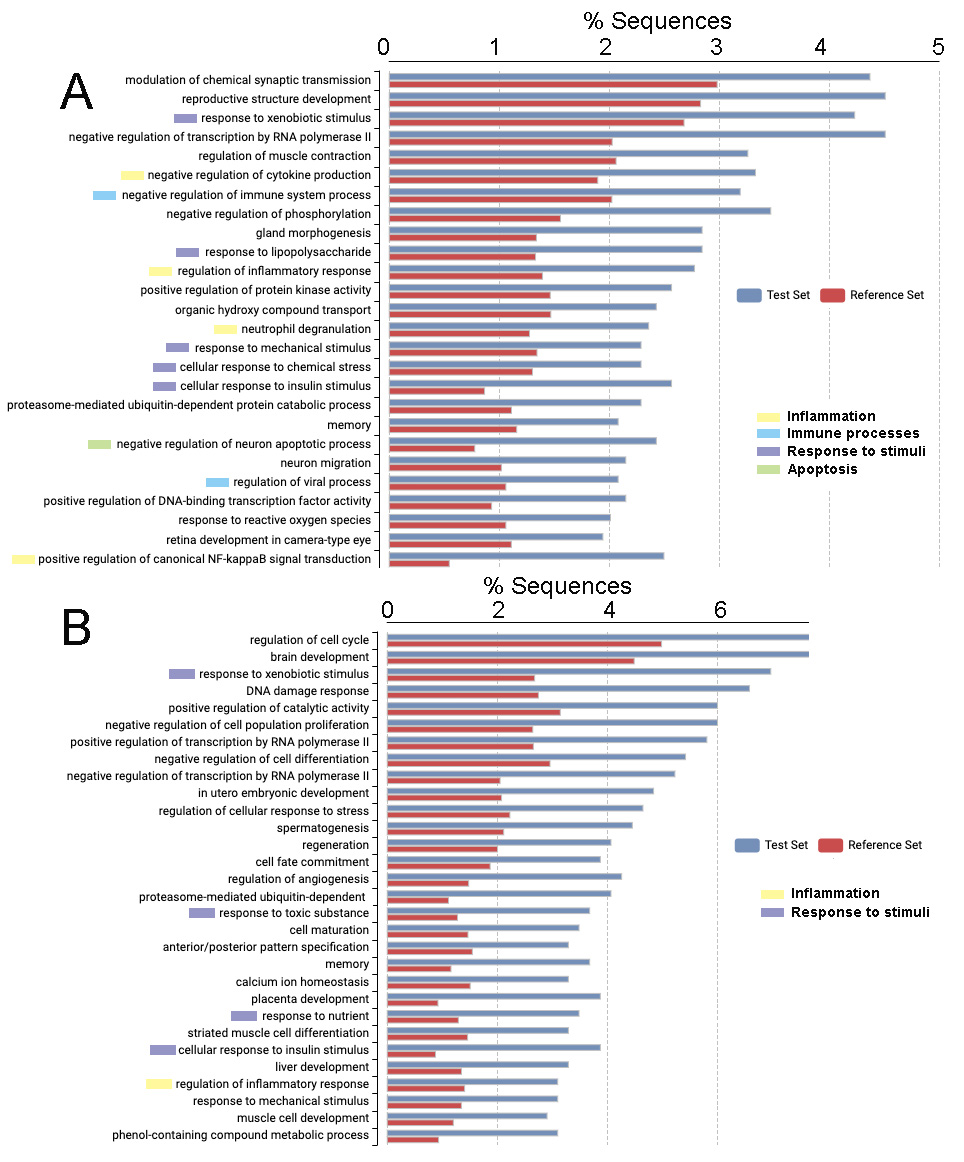


Supplementary Figure 8. Enrichment analysis showing the modulated processes in internal mussel immune barriers. (A) Biological enriched process in gills after the bacterial waterborne infection. (B) Biological enriched process in hemolymph hemocytes after the bacterial waterborne infection.

Supplementary Table 1. Reference databases for taxonomy profiling.

| **Taxonomic group** | **Number of reference genomes/sequences** | **Source** |
| --- | --- | --- |
| **Algae** | 320 | RefSeq |
| **Protozoa** | 93 | RefSeq |
| **Fungi** | 388 | RefSeq |
| **Bacteria** | 5,396 | CLC Workbench Curated Database |
| **Virus** | 28,021 | C-Reference Viral Database (RVDB)-NCBI Viral Genomes |
| **Annelida** | 37 | RefSeq |
| **Cnidaria** | 11 | RefSeq |
| **Mollusca** | 17 | RefSeq |
| **Fish** | 3 | RefSeq |
| **Crustacea** | 6 | RefSeq |
| **Echinodermata** | 6 | RefSeq |
| **Nematoda** | 306 | RefSeq |
| **Platyhelminthes** | 91 | RefSeq |
| **Porifera** | 7 | RefSeq |

Supplementary Table 2. Primers used for qPCR validation of apoptosis resistance in ISCs.

| **Name** | **Sequence (5´- 3´)** | **Efficiency** |
| --- | --- | --- |
| **Caspase 1** | For: GATCTTGGAAGTGGTGTAGAACG | -3.42 |
|  | Rev: CACTGCTAGGAAATCTGCTTCAT |  |
| **Caspase 2** | For: GATATATGACAAGGGTGGCAATG | -3.24 |
|  | Rev: GACTTTACAGCATCCAGGACATC |  |
| **Caspase 3/7** | For: CAATGTGTAAAAACGAGAGACATTG | -3.43 |
|  | Rev: GTTAGTATATGCCCACTGTCCATTC |  |
| **Caspase 8** | For: CCCAACCAGTAGTAACACCAGAC | -3.3 |
|  | Rev: GTATGAACCATGCCCCTATATCA |  |
| **Bcl2** | For: AGATAACGGTGGTTGGCAAG | -3.05 |
|  | Rev: TAACGCCATTGCGCCTAT |  |
| **Bax2** | For: CCAACAGGTCCACCATTAGAAC | -3.19 |
|  | Rev: CTCTTGGCCACAGTTAGGAATG |  |
| **18S** | For: GTACAAAGGGCAGGGACGTA | -3.3 |
|  | Rev: CTCCTTCGTGCTAGGGATTG |  |

Supplementary Table 3. Differentially expressed genes.

Additional Excel File

Supplementary Table 4. Sequencing information 4. The Mg3 matches column shows the number of reads matching the mussel genome, and the Mg3 % of matches column shows the percentage.

| **Samples** | **Raw sequences** | **Mg3 matches** | **Mg3 % of matches** |
| --- | --- | --- | --- |
| Hemolymph hemocytes_Control_1 | 20,282,212 | 17,640,526 | 86.98 |
| Hemolymph hemocytes_Control_2 | 23,622,390 | 20,318,544 | 86.01 |
| Hemolymph hemocytes_Control_3 | 21,600,168 | 18,423,330 | 85.29 |
| Intervalvar hemocytes_Control_1 | 29,719,986 | 21,378,210 | 71.93 |
| Intervalvar hemocytes_Control_3 | 23,634,438 | 10,916,926 | 46.19 |
| Intervalvar hemocytes_Control_5 | 21,392,624 | 6,306,124 | 29.48 |
| Gills_Control_1 | 23,385,740 | 20,060,866 | 85.78 |
| Gills_Control_2 | 24,010,546 | 19,777,594 | 82.37 |
| Gills_Control_3 | 25,168,132 | 20,685,432 | 82.19 |
| Hemolymph hemocytes_Infected_1 | 24,147,000 | 20,975,332 | 86.87 |
| Hemolymph hemocytes_Infected_2 | 22,959,082 | 21,061,196 | 91.73 |
| Hemolymph hemocytes_Infected_3 | 23,114,084 | 19,891,790 | 86.06 |
| Intervalvar hemocytes_Infected_3 | 23,432,766 | 8,723,248 | 37.23 |
| Intervalvar hemocytes_Infected_4 | 23,861,624 | 9,196,626 | 38.54 |
| Intervalvar hemocytes_Infected_6 | 32,422,794 | 25,074,302 | 77.34 |
| Gills_Infected_1 | 22,200,928 | 18,032,632 | 81.22 |
| Gills_Infected_2 | 26,018,524 | 21,223,928 | 81.57 |
| Gills_Infected_3 | 22,904,638 | 18,806,556 | 82.11 |
